# Expansion of the Emerging Fungal Pathogen *Cryptococcus bacillisporus* Into America: Linking Phylogenetic Origin, Geographical Spread and Population Under Exposure Risk

**DOI:** 10.3389/fmicb.2020.02117

**Published:** 2020-08-28

**Authors:** Jorge G. Carvajal, Alberto J. Alaniz, Mario A. Carvajal, Emily S. Acheson, Rodrigo Cruz, Pablo M. Vergara, Massimo Cogliati

**Affiliations:** ^1^Facultad Tecnológica, Universidad de Santiago de Chile, Santiago, Chile; ^2^Facultad de Ingeniería, Universidad de Santiago de Chile, Santiago, Chile; ^3^Department of Geography, The University of British Columbia, Vancouver, BC, Canada; ^4^Laboratorio de Micología, Universidad de Valparaíso, Valparaíso, Chile; ^5^Dipartimento di Scienze Biomediche per la Salute, Università degli Studi di Milano, Milan, Italy

**Keywords:** *Cryptococcus bacillisporus*, molecular type VGIII, Chile, niche modeling, MLST

## Abstract

In 2018 the fungal pathogen *Cryptococcus bacillisporus* (AFLP5/VGIII) was isolated for the first time in Chile, representing the only report in a temperate region in South America. We reconstructed the colonization process of *C. bacillisporus* in Chile, estimating the phylogenetic origin, the potential spread zone, and the population at risk. We performed a phylogenetic analysis of the strain and modeled the environmental niche of the pathogen projecting its potential spread zone into the new colonized region. Finally, we generated risk maps and quantified the people under potential risk. Phylogenetic analysis showed high similarity between the Chilean isolate and two clonal clusters from California, United States and Colombia in South America. The pathogen can expand into all the temperate Mediterranean zone in central Chile and western Argentina, exposing more than 12 million people to this pathogen in Chile. This study has epidemiological and public health implications for the response to a potential *C. bacillisporus* outbreak, optimizing budgets, routing for screening diagnosis, and treatment implementation.

## Author Summary

Fungal diseases have been neglected over the last years, being cryptococcal disease a cause of high impact in global health. Here we determined the phylogenetic origin of the first *C. bacillisporus* strain found in a veterinary isolation in Chile which was identified as a new multi-locus sequence typing of a VGIII genotype closely related to two clonal clusters from California United States, and Colombia. Then, we found that the pathogen can expand into all the temperate Mediterranean zone of central Chile and western Argentina from 29.5° to 36.6° south latitude. Although no human case of cryptococcosis by *C. bacillisporus* have been reported in Chile, in last decades this pathogen has emerged in locations where veterinary cases were reported, affecting both immunocompromised and immunocompetent humans. For this reason, we generated risk maps and quantified the population under different exposure risk levels, finding that more than 12 million people are in risk in Chile, which 74% of them are in a very high risk level.

## Introduction

The pathogenic *Cryptococcus* species complexes, *Cryptococcus neoformans* and *Cryptococcus gattii*, are the etiological agents of cryptococcosis, an invasive fungal disease with a high mortality rate and high public health costs worldwide (Mourad, 2018). These fungi generate different clinical and epidemiological consequences, which depend mainly on the immunity of the host and the pathogenicity of the agent (Rajasingham et al., 2017; George et al., 2018). The *C. gattii* species complex includes basidiomycetous yeasts that can be isolated from different environmental sources such as soil, decaying organic material and tree hollows, as well as from infected human and animal hosts where they grow as asexual budding yeasts (Cogliati, 2013). During sexual reproduction, this fungus presents a hyphal growth generating basidiospores which, together with asexual blastospores, play an important role as infectious propagules being dispersed by wind and then inhaled by hosts (Velagapudi et al., 2009; Voelz et al., 2013; You and Xu, 2018). Unlike cryptococcosis caused by the yeasts belonging to the *C. neoformans* species complex, which affects mostly immunocompromised hosts, *C. gattii* species complex strains also have the capacity to affect immunocompetent hosts as reported by recent studies (Mcmullan et al., 2013; Chen et al., 2014; Li et al., 2020). The *C. gattii* species complex has largely been historically restricted to tropical and subtropical regions, however records from 1990 in San Francisco California (United States), suggest their expansion into temperate zones in America (Pfeiffer and Ellis, 1991; Acheson et al., 2019). Since then, the most important outbreaks in the temperate zone were British Columbia Canada and the Northwest Pacific coast of the United States both in 1999, affecting immunocompromised and immunocompetent hosts and generating a public health alarm (Espinel-ingroff and Kidd, 2015). The species complex can be subdivided into two serotypes, B and C, based on the capsular antigenic pattern, and into five species named *Cryptococcus gattii* (VGI), *Cryptococcus deuterogattii* (VGII), *Cryptococcus bacillisporus* (VGIII), *Cryptococcus tetragattii* (VGIV), *Cryptococcus decagattii* (VGIV/VGIIIc) depending on molecular typing using different techniques (Boekhout et al., 2001; Meyer et al., 2003; Meyer et al., 2009; Feng et al., 2013; Cogliati et al., 2014; Firacative et al., 2016; Hagen et al., 2015). In addition, a further putative cryptic species denoted VGV has been recently identified (Farrer et al., 2019). However, this new taxonomy needs to be further validated and it is not yet accepted by the whole scientific community (Kwon-Chung et al., 2017). In North America, the preponderant species from clinical, veterinary, and environmental isolates are *C. deuterogattii* and *C. bacillisporus*. The former, *C. deuterogattii*, was identified in British Columbia, Canada and in Washington and Oregon in the United States (Kidd et al., 2004; Byrnes and Marr, 2011). By comparison, *C. bacillisporus* (VGIII) was identified in patients with HIV/AIDS in the United States, especially in Southern California, with fewer cases in Oregon, Washington, New Mexico, New Jersey, Michigan, and Alaska. Outside the United States, infections were reported in Latin America from Mexico, Colombia, Brazil, Paraguay, Argentina, Venezuela, and Guatemala (Byrnes et al., 2011; Walraven et al., 2011; Lockhart et al., 2013; Lizarazo et al., 2014; Singer et al., 2014; Springer et al., 2014; González et al., 2016; Vélez and Escandón, 2017; Berejnoi et al., 2019). However, a recent veterinary isolation in a domestic animal was reported for the first time in central Chile, in the locality of Limache, 15 kilometers south of the city of Valparaíso (30°59′S, 71°17′W), extending its southern geographical limit to a geographical area where it has never been recorded before (non-endemic species) (Vieille et al., 2018). This finding has generated concern in Chilean public health institutions and among researchers, mainly because this is the first time that a *C. bacillisporus* has been recorded in another American temperate zone. The similar climate conditions between Chile’s Mediterranean zone and California suggests a potential dispersal of the pathogen favored by similar climatic conditions and hence possible environmental suitability. Previous studies have identified the spatial distribution of *C. neoformans* and *C. gattii* species complex distributions in Europe through the ecological niche modeling of their bioclimatic conditions suitable for their survival (Cogliati et al., 2017; Alaniz et al., 2020), methodology that has also been succesfully applied to other recent global infectious diseases (Alaniz et al., 2017, 2018). Considering the latter, the aim of this study is to analyze: (i) the geographical and phylogenetic origin of the sample; (ii) the potential geographical spread in this new area; and (iii) the population exposed to the risk. The achievement of these objectives may contribute to generate focused surveillance and preventive actions that could help to understand the potential negative health consequences in the population.

## Materials and Methods

### Geographical Origin, Source, and Identification of the Chilean *C. bacillisporus* Isolate

The first autochthonous *C. bacillisporus* isolate (VGIII) was recently identified in Chile from a nasal injury in a domestic cat (Vieille et al., 2018). Mucocutaneous cryptococcosis was suspected in the animal, so nasal samples were taken to perform mycological studies. The nasal swab was analyzed in the Mycology Laboratory of the Universidad de Valparaíso, where the sample was cultured on Sabouraud dextrose agar under optimal conditions (37°C) obtaining encapsulated yeast cells after 48 h. The sample was sub-cultured on Staib agar, urea agar, and canavanine-glycine-bromothymol blue agar, and incubated at 30°C which, after 7 days, led to the classification of the yeast as *C. gattii* species complex. The isolate was then identified as *C. bacillisporus*, molecular type VGIII, by restriction fragment length polymorphism of *URA5* and confirmed by duplex PCR (Meyer et al., 2003; Feng et al., 2013). Mating type allelic pattern αC was determined by multiplex PCR as described elsewhere (Cogliati et al., 2014).

### Phylogenetic Analysis

To analyze the possible origin of the new Chilean *C. bacillisporus* isolate, we performed a phylogenetic analysis through the standardized genotyping technique called multi locus sequence typing (MLST) (Meyer et al., 2009), applied using the standard ISHAM MLST scheme by sequencing seven house-keeping loci (*CAP59*, *GPD1*, IGS1, *LAC1*, *PLB1*, *SOD1*, *URA5*) (Meyer et al., 2009). Allele types for each locus and the sequence type (ST), determined by the combination of the seven allele types, were assigned by matching the sequences with those present in the *C. gattii* MLST database^1^. A phylogenetic tree was reconstructed comparing the ST of the Chilean isolate with 69 different STs from VGIII global isolates previously reported (Byrnes et al., 2011; Lizarazo et al., 2014; Singer et al., 2014; Springer et al., 2014; González et al., 2016). Analysis was performed by MEGA software v6.06^2^ using maximum likelihood algorithm, Tamura-Nei model, nearest-neighbor-interchange method starting from a neighbor-joining tree for tree inference, and bootstrap analysis on 1000 random repeats as phylogeny test. Allele type combinations were also input in Phyloviz software v2.0^3^ to construct a minimum spanning tree in order to recognize clonal clusters among VGIII STs as well as the most probable founder genotypes.

### Potential Geographical Spread in Chile

In order to predict the distribution range of *C. bacillisporus*, we used species distribution models (SDM) based on the maximum entropy algorithm with MaxEnt 3.4.1k software (Phillips et al., 2006). MaxEnt requires two types of input data: occurrence points of the organisms and environmental variables which constitute the predictors. The aim is to predict the environmental suitability for the species based on its ecological niche requirements. The spatial prediction of an SDM can be homologated to the potential abundance of organisms (Phillips et al., 2006), and it has proven to generate reliable results for the modeling of infectious disease vectors (Alaniz et al., 2017, 2018, 2020). We compiled and systematized an occurrence database consisting of 29 occurrences of *C. bacillisporus* environmental isolates according to the following criteria: (A) avoiding isolates from humans due to the uncertainty related to the movement of the patient, however we included four human occurrences from Lockhart et al., 2013 which denoted no travel history of patients, (B) excluding those without specific geographical coordinates (latitude and longitude) reducing the uncertainty on the location and (C) excluding the Chilean isolate to predict the distribution based on the source zone niche (Supplementary Appendix Figure S1 and Supplementary Appendix Table S1) (Pfeiffer and Ellis, 1991; Escandón et al., 2006; Mazza et al., 2013; Singer et al., 2014; Springer et al., 2014; Espinel-ingroff and Kidd, 2015). The environmental variables used as predictors of the spatial distribution model consisted of the bioclimatic layers of the WorldClim 2 project^4^ with 2.5 arc minutes spatial resolution (approximately 5 km × 5 km cells) (Fick, 2017), plus relative humidity (RH), enhanced vegetation index (EVI), mean annual solar radiation (SR), topographic diversity index (TDI), vegetation continuous fields (VCF), net primary productivity (NPP), mean annual wind speed, elevation, soil bulk density, soil organic carbon at 0–15 cm depth and mean soil pH at 0–15 cm depth (Supplementary Appendix Table S2). All the remote sensing variables such as VCF, TDI, NPP, and EVI were processed in Google Earth Engine Platform (Gorelick et al., 2017). To improve the quality of the data used for the modeling process, we carried out two measures. First, we reduced the spatial autocorrelation and geographical bias of the occurrences dataset by applying a spatial rarefy function in a GIS environment, retaining only the occurrences that were separated by at least 15 km. Second, we reduced the collinearity between predictor variables by generating a preliminary model with the complete set of variables. According to the percent contribution of each variable combined with a correlation matrix using the Shapiro–Wilk (normality distribution) test and Spearman (correlation) test, we excluded variables with a low percent of contribution and a high correlation coefficient (more than ± 0.7 in the correlogram). The final model was constructed using a bootstrap resampling method and retaining the values according to the 95% confidence interval. The accuracy of the model was assessed from the area under the curve (AUC) of the receiver operating characteristic, which estimates the sensitivity and specificity of the model by partitioning the occurrences dataset into a training and a test dataset, the latter used exclusively for model evaluation. Finally, we used the habitat suitability predicted by the model in Chile to estimate the potential spread of the *C. bacillisporus.* In the already occupied zones of the United States, we considered the suitability predicted by the model as the geographic distribution of the pathogen.

### Estimation of the Population Under Exposure Risk

To estimate the risk, we included two components: (i) the potential abundance of the pathogen (threat); and (ii) the human population density (vulnerability). To assess the risk, we used human population density grid (version 4 of the year 2020) with a spatial resolution of 2.5 arc minutes from Socioeconomic Data and Application Centre of NASA^5^. We classified human population density in four levels: null (0–1 inhabitants/km^2^), low (>1–10 inhabitants/km^2^), medium (>10–100 inhabitants/km^2^), and high (>100 inhabitants/km^2^). Then, we assigned a numerical value to each of these categories (null = 0, low = 1, medium = 2, high = 3). On the other hand, we reclassified the suitability map of *C. bacillisporus* into four levels (null, low, medium, and high). The null level corresponded to the non-significant suitability value, considering as threshold the values under the 10th percentile. Then, the significant suitability values (>10th percentile) were divided into three categories considering equal intervals. We assigned a numeric value to each one of these suitability categories (null = 0, low = 1, medium = 2, and high = 3). Finally, we used a double entry matrix to multiply both reclassified rasters (Supplementary Appendix Figure S2) (population density and suitability) in a geographic information system. As product we obtained a map of potential exposure risk with five risk levels: very low, low, medium, high, and very high (Alaniz et al., 2017).

To quantify population at different risk levels we used the population count by square kilometer of NASA, which was overlapped with the generated exposure risk map. Population count product corresponds to an estimation of population per pixel based on national and subnational censuses and projected to the year 2020 (Doxsey-Whitfield et al., 2015). We quantified the population under risk exposure in both colonized (Chile) and the potential source (United States) zones, considering subnational administrative division per level of risk. For the product, we generated a series of maps and tables showing the population under risk per subnational administrative division and level of risk.

## Results

### Chilean Isolate Originated From One of the Two Major VGIII Clusters

**FIGURE 1:**
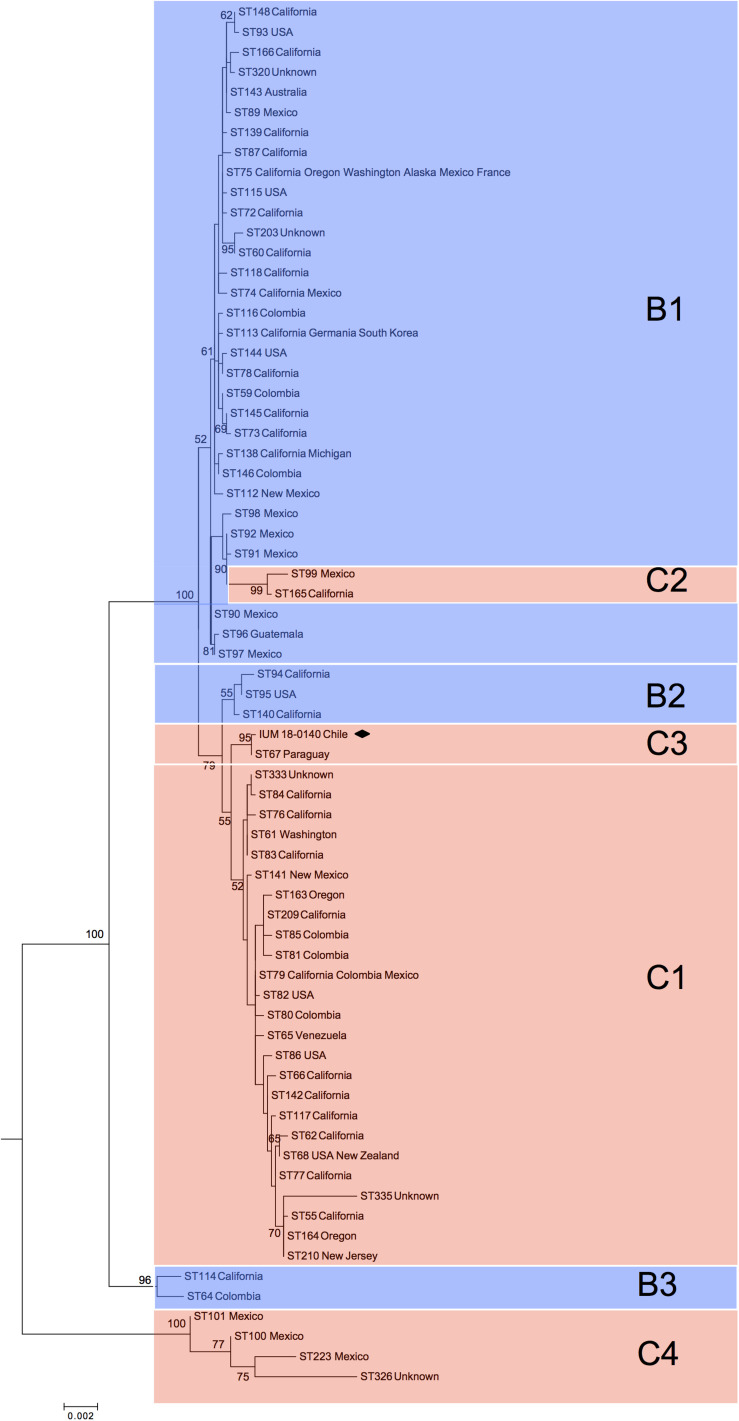
Phylogenetic reconstruction by maximum likelihood method resulting from the comparison of the Chilean isolate (black square) with 69 global *Cryptococcus bacillisporus* (VGIII) STs. C1, C2, C3, and C4 identify clusters including serotype C strains, while B1, B2, and B3 identify clusters including serotype B strains. Results of bootstrap analysis (>50%) are indicated beside each node.

Phylogenetic analysis of global *C. bacillisporus* isolates showed the presence of two major clusters reported as B1 and C1 (Figure 1), and further five minor clusters (B2, B3, C2, C3, and C4). Cluster B1 included 31 STs belonging to serotype B isolates mainly from United States and Mexico, cluster B2 included 3 STs from United States, and B3 grouped two phylogenetically distant STs (ST114 and ST64). Cluster C1 included 25 STs belonging to serotype C isolates from North and South America, cluster C2 was represented by two STs of isolates from Mexico and United States, and C4 grouped four STs from Mexico which are genetically very distant from the other VGIII STs. The Chilean *C. bacillisporus* (VGIII) isolate presented a new MLST profile (ST552: *CAP59*-20, *GPD1*-23, IGS1-112, *LAC1*-23, *PLB1-*23, *SOD1*-29, *URA5*-21), which did not match any of the profiles in the MLST database. Observing the phylogenetic tree, it was included in cluster C3, genetically related to cluster C1, together with an isolate from Paraguay belonging to genotype ST67, differing in IGS1 and *PLB1* loci presenting one single nucleotide polymorphism each.

**FIGURE 2:**
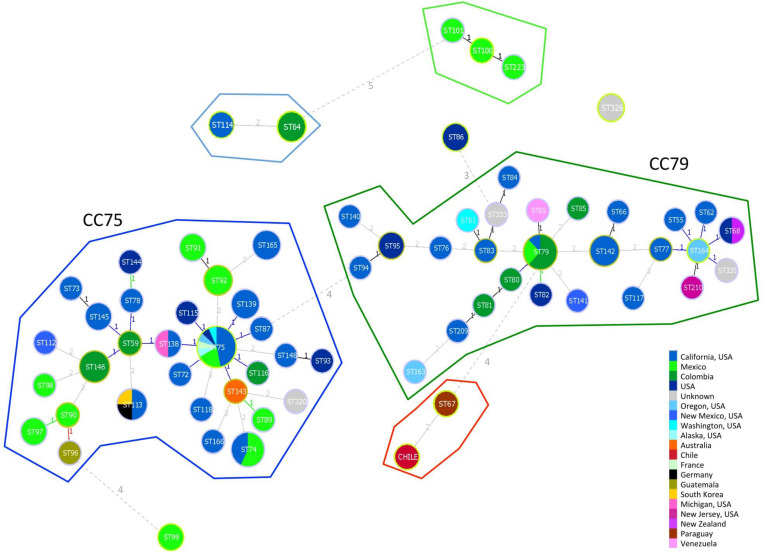
Minimum spanning tree reporting the main clonal clusters among *C. bacillisporus* VGIII sequence types. Circle sizes are proportional to the number of strains with the same sequence type. Different colors represent different geographic origins. Numbers beside the lines linking two circles indicate the number of different alleles. Yellow circles surrounding STs identify the founder of a clonal cluster. Clonal clusters, including STs differing in one or two alleles, are grouped inside closed lines with different colors.

The minimum spanning tree showed a similar picture with two main clonal clusters including most of the VGIII STs (Figure 2). Clonal cluster CC75 grouped 30 STs probably all originated from ST75 shared by a large number of VGIII isolates from the United States and Mexico. The probable founder for the second clonal cluster (CC79) was ST79, which was shared by both South and North American isolates and gave rise to the small cluster containing the Chilean and Paraguayan isolates.

### *Cryptococcus bacillisporus* Potentially Colonizing Central Chile Region

The generated model reached an AUC = 0.979 ± 0.009 (Supplementary Appendix Figure S3), showing less than 20% uncertainty in the areas with the highest predicted suitability values (United States and Chile) (Supplementary Appendix Figure S4). The variables that mostly contributed to the final model were precipitation in the warmest quarter, precipitation in the coldest quarter, SR, and EVI, with PCs of 51.2, 22.1, 10.2, and 4.4%, respectively (Supplementary Appendix Figure S5 and Supplementary Appendix Table S3). The fungus has a suitability peak at 0 mm of precipitation in the warmest quarter, the same pattern observed for the precipitation of the coldest quarter variable, but with a peak at 350 mm. In the case of SR, we identified a suitability peak at 18,000 kJ m^–2^ day^–1^. Finally, in the case of EVI, the suitability presented a Gaussian pattern with a maximum value at 0.23.

**FIGURE 3:**
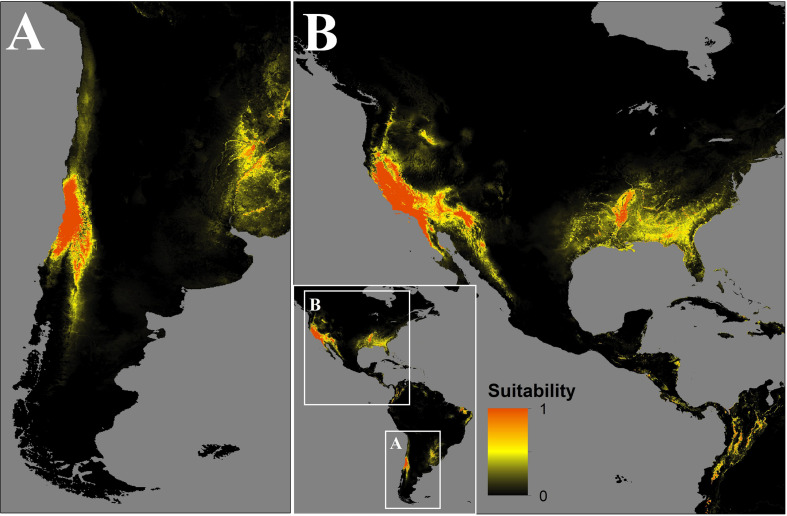
Map of the distribution of *C. bacillisporus* predicted by MaxEnt niche modeling in South **(A)** and North America **(B)**.

In Chile, the predicted spread could occupy the central-north zones of Chile from 29.5°S to 36.6°S, mainly in the Coquimbo, Valparaíso, Santiago, O’Higgins, and El Maule regions, as well as the Andean boundary with Argentina until 40°S (Figure 3A).

In the source zone, the distribution of *C. bacillisporus* is mainly located in the west coast of the United States, mainly in California and Arizona, as well as in the northwest coastal zone of Mexico. On the other hand, in the east coast of the United States, Mississippi, Arkansas, Alabama, Georgia, South Carolina, Florida, and Louisiana showed high suitability for *C. bacillisporus*, but lower than the west coast (Figure 3B).

### Potentially Exposed Population

**FIGURE 4:**
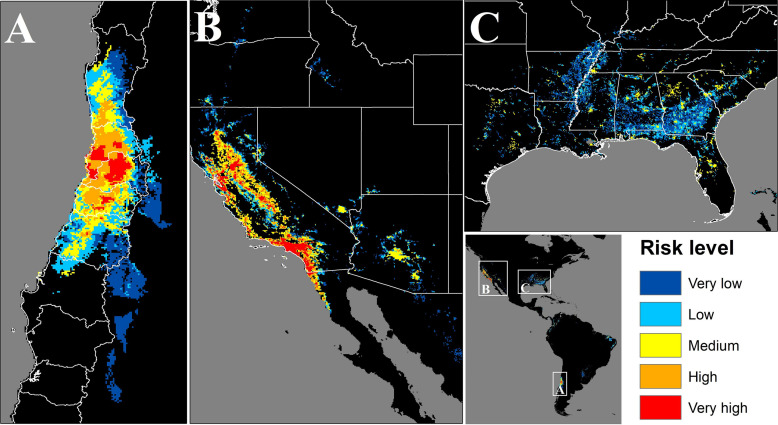
Map of the areas where population could be potentially exposed to a significant risk of contact with *C. bacillisporus* in Chile **(A)**, Western United States **(B)**, and Eastern United States **(C)**. CQ = Coquimbo, VP = Valparaiso, ST = Santiago Metropolitan region, OH = Libertador Bernardo O’Higgins, MA = Maule, BB = Biobío, CA = California, NV = Nevada, OR = Oregon, WS = Washington, CO = Colorado, ID = Idaho, AZ = Arizona, TX = Texas, LA = Louisiana, AR = Arkansas, MO = Missouri, MS = Mississippi, AL = Alabama, GA = Georgia, FL = Florida, TN = Tennessee, NC = North Carolina, SC = South Carolina.

In Chile the potentially exposed population reached 12,298,551 people, of which 12.8% and 74% correspond to high and very high exposure risk levels, respectively. The population exposed to a significant risk level (above medium), represents 56.7% of the total Chilean population by the year 2020. The most affected administrative regions are the Santiago Metropolitan, Valparaíso, and Libertador Bernardo O’Higgins regions, with 94, 83.3, and 81.3%, respectively, of their total population exposed to a significant risk level (Figure 4A and Table 1). Finally, the Maule, Coquimbo, and Biobío administrative regions have 42.4, 23.2, and 7.8% of their total population exposed to significant risk levels (Figure 4A and Table 1). The population per risk level and province is detailed in Supplementary Appendix Table S4.

**TABLE 1:** Population per risk level and region in Chile.

**Region**	**Very low**	**Low**	**Medium**	**High**	**Very high**	**Total at risk**	**Total population**	**%**
Santiago	5170	5891	80591	449288	7692318	8233258	8745008	94.0
Valparaíso	907	56721	43029	392903	1289325	1782885	2070962	83.3
O’Higgins	244	87636	233478	502846	85200	909405	1010578	81.3
Maule	42068	266052	242769	207871	34590	793350	1143959	42.4
Coquimbo	32689	113127	182739	24440	0	352994	893963	23.2
Biobío	564	57065	169030	0	0	226659	2174734	7.8

*Total population at risk, total population per region to the year 2020, and percentage of population at risk are presented.*

In the source zone, the population under exposure risk reached 63,521,158 people, of which 5.9 and 47.5% corresponds to high and very high exposure risk levels. On the other hand, 41.3% of the population is exposed to medium risk level, and only 4.3 and 0.9% are under low and very low levels, respectively. In the United States, California, Nevada, and Arizona are the most affected states, with 86, 76.2, and 64.6%, respectively of their total population exposed to significant risk levels. On the other hand, Alabama, Georgia, Mississippi, and South Carolina have more than 20% of their total population exposed to significant risk level, while Tennessee, Florida, Louisiana, and Arkansas have more than 12%. Finally, thirteen states have less than 8% of their total population exposed to risk level above medium (Figures 4B,C and Table 2). The population per risk level and district is detailed in Supplementary Appendix Table S5.

**TABLE 2:** Population per risk level and region in the United States.

**State**	**Very low**	**Low**	**Medium**	**High**	**Very high**	**Total at risk**	**Total population**	**%**
California	49745	280585	1396534	3635888	30139839	35502591	40892618	86.0
Nevada	6108	40334	2782162	12700	3142	2844445	3670789	76.2
Arizona	24626	141256	5144344	107111	4490	5421827	8129861	64.6
Alabama	89537	461879	1848695	0	0	2400111	5157663	35.8
Georgia	149659	579610	3501758	17211	0	4248238	11750113	29.9
Mississippi	52656	209572	753992	6699	0	1022919	3108633	24.5
South Carolina	13743	179500	1138241	2908	0	1334392	5269431	21.7
Tennessee	14652	88894	1297949	0	0	1401495	7096093	18.3
Florida	34072	252144	3243158	1908	0	3531282	21468401	15.1
Louisiana	25132	89555	668598	321	0	783606	4714576	14.2
Arkansas	60004	139222	410394	0	0	609620	3244168	12.7
Texas	14058	100071	2460046	75	0	2574250	30909075	8.0
Puerto Rico	1	0	266908	0	0	266909	3554936	7.5
North Carolina	877	55738	739671	0	0	796286	11348378	6.5
Oregon	4467	32526	157892	9504	0	204390	4307474	3.9
Kentucky	6437	22452	142806	0	0	171694	4788476	3.0
Utah	796	13312	92759	0	0	106867	3454124	2.7
Indiana	630	4989	104817	0	0	110435	6908307	1.5
Missouri	18236	45230	58448	0	0	121914	6436280	0.9
Idaho	4778	10289	8626	0	0	23693	1931340	0.4
Washington	3705	6240	9912	1631	0	21488	7540985	0.2
Illinois	2765	7052	7576	0	0	17392	13153406	0.1
Oklahoma	1003	1372	0	0	0	2375	4094597	0.0
Virginia	552	2387	0	0	0	2939	9061400	0.0

*Total population at risk, total population per region to the year 2020, and percentage of population at risk are presented.*

## Discussion

### Assumptions and Limitations

This study incorporates some main assumptions and uncertainties which are necessary to discuss. First, the methodology used to predict the potential distribution of *C. bacillisporus* was based on 29 isolations from the genetically identified potential source zone (California and Colombia), not including the occurrence recorded from Chile. We decided to not include the Chilean occurrence to test if the high-risk zone predicted by the model overlaps with this record. We identified that the occurrence recorded in Chile (Limache at 30°59′S, 71°17′W) coincides with the high risk zone predicted by the model, confirming its reliability. However, since there is only one occurrence record for Chile, it is needed to further verify in the time and space by developing random environmental surveys if the fungus is actually present in the complete predicted high risk range. In this context our study represents a baseline to analyze the potential arrival, spread and establishment of *C. bacillisporus* in Chile, presenting a possible scenario that needs to be monitored and tracked in the light of this first isolation that could represent the actual arrival of the pathogen. Second, our prediction is based on the Niche Conservatism Principle which assumes that the species maintains they environmental niche requirements in the colonized zone (Gallien et al., 2012). However, non-native alien species can increase their distributional ranges in the colonized zone (non-stability hypothesis), thus our results could underestimate the potential distribution range (Broennimann et al., 2007). Third, it is also important to identify at species and molecular type level all isolates from cases of cryptococcosis to verify if they correspond to infections due to *C. bacillisporus*. Fourth, a potential source of uncertainty for our study could be related with the four clinical occurrences included from Lockhart et al. (2013), in which it is possible that some of those patients got infected in states other than the ones in which they were recorded. However, these occurrences represent only the 13.8% of the complete dataset.

### Insights and Implications

In the present study, we examined the potential role of *C. bacillisporus* as emerging pathogen in Chile. Even when this pathogen has never been documented to infect a human in this country, and our study is based on the single isolation of the fungus in a veterinary case, a possible outbreak cannot be ruled out. During the last decades *C. bacillisporus* has emerged as a cause of disease in immunocompromised humans in southern California, United States, areas where according epidemiological studies cats are most often infected with *C. bacillisporus* compared with other animals (Trivedi et al., 2011; Singer et al., 2014).

Two main genotypes, ST75 and ST79, were identified as founders of two large phylogenetically distinct clonal clusters containing the majority of the globally identified STs. The presence of two VGIII phylogenetic clusters, one including serotype B and one serotype C isolates, were previously reported by other authors (Meyer et al., 2003; Byrnes et al., 2011; Walraven et al., 2011; Lockhart et al., 2013; Singer et al., 2014; Springer et al., 2014). The majority of STs included in the CC75 were from North America particularly from California United States, while the ones grouped in the CC79 included several STs identified in South America, particularly from Colombia. This is in agreement with a recent phylogenetic study carried out using whole genome sequencing of a set of VGIII global isolates, which describes Mexico and the United States as the origin of the VGIII serotype B population, and Colombia as the origin of serotype C population (Firacative et al., 2016). The phylogenetic analysis grouped the Chilean isolate (C3) with a strain from Paraguay, which suggests a close link to the genotype ST79 related to Colombia (Figure 2) in the CC79 cluster. There is also possible that the populations from CC79 cluster could have migrated north, stablishing in North America and further developing the CC75 cluster, as it happened before with the spread of VGII molecular type from South American Amazonian Rainforests to North America and Columbia, Canada (Chen et al., 2014; Souto et al., 2016; Engelthaler and Casadevall, 2019).

The spatial methodological framework presented here was successfully used in a recent study to determine the risk zones in Europe to different cryptococcal pathogens (Alaniz et al., 2020). The suitable geographic areas for *C. bacillisporus* survival are characterized by scarce or null rainfall during the dry season and moderate rainfall during the cold season, high SR and scarce vegetation corresponding to the warm summer Mediterranean climate present in California and northwestern Mexico (Acheson et al., 2019). The same climatic conditions are present in Central Chile, where the *C. bacillisporus* isolate was recovered, suggesting that VGIII is spreading outside the endemic areas and is potentially able to colonize a wide area in Chile covering about 150,000 km^2^. The same pattern of colonization has been present in other organisms that colonized central Chile, reinforcing the results presented here (Carvajal et al., 2019). This area corresponds to the most populated area of the country, including the capital city of Santiago. The colonization of the whole area by *C. bacillisporus* would expose more than 12 million people to contact with this pathogen, posing a serious risk for sensitive categories such as patients living with HIV, which in Chile has a prevalence of 0.34%, of whom only one half has access to antiretroviral therapy^6^.

On the other hand, the information generated here may provide guidance for decision making about this pathogen in the United States, which can be highly valuable considering the large amount of people under exposure risk. The generated maps can represent a tool for the design of contingency plans, public health strategies, budget optimization and routing for screening, diagnosis and treatments in the high-risk zones.

This study supplies basilar information for monitoring the evolution of *C. bacillisporus* epidemiology in Chile. The potential distribution map of the pathogen as well as the risk map reported here represent an important tool to plan future environmental surveys and clinical screening of patients exposed to the pathogen in the high risk areas. Identification of limited geographical areas at high risk allows for punctual intervention of public health, avoiding dispersion and waste of scarce and highly valuable health resources. Finally, the framework used in this study can be applied to other environmentally driven pathogens, helping to foresee the impacts and repercussions in public health before the actual potential spread. The linkage of phylogenetic analysis with ecological niche modeling allows to generate a new level of precision and accuracy in the prediction of pathogen distributions.

## Data Availability Statement

The raw data supporting the conclusions of this article will be made available by the authors, without undue reservation.

## Ethics Statement

The present study analyzed data already reported and published by other authors and thus it does not require an approval from an ethical committee.

## Author Contributions

All authors listed have made a substantial, direct and intellectual contribution to the work, and approved it for publication.

## Conflict of Interest

The authors declare that the research was conducted in the absence of any commercial or financial relationships that could be construed as a potential conflict of interest. The reviewer KF-P declared a past collaboration with one of the authors MC to the handling editor.

## Footnotes

1 www.mycologylab.org

2 www.megasoftware.net

3 www.phyloviz.net

4 www.worldclim.org

5 https://sedac.ciesin.columbia.edu

6 https://www.unaids.org/en/regionscountries/countries/chile
