# Supplementary material for: Expansion of the Emerging Fungal Pathogen Cryptococcus bacillisporus Into America: Linking Phylogenetic Origin, Geographical Spread and Population Under Exposure Risk
Source: Front Microbiol. 2020 Aug 28;11:2117. doi: 10.3389/fmicb.2020.02117 (PMC7485214; doi:10.3389/fmicb.2020.02117)
Supplement: Supplementary file 1 [file Table_1.DOCX]

**Appendices**


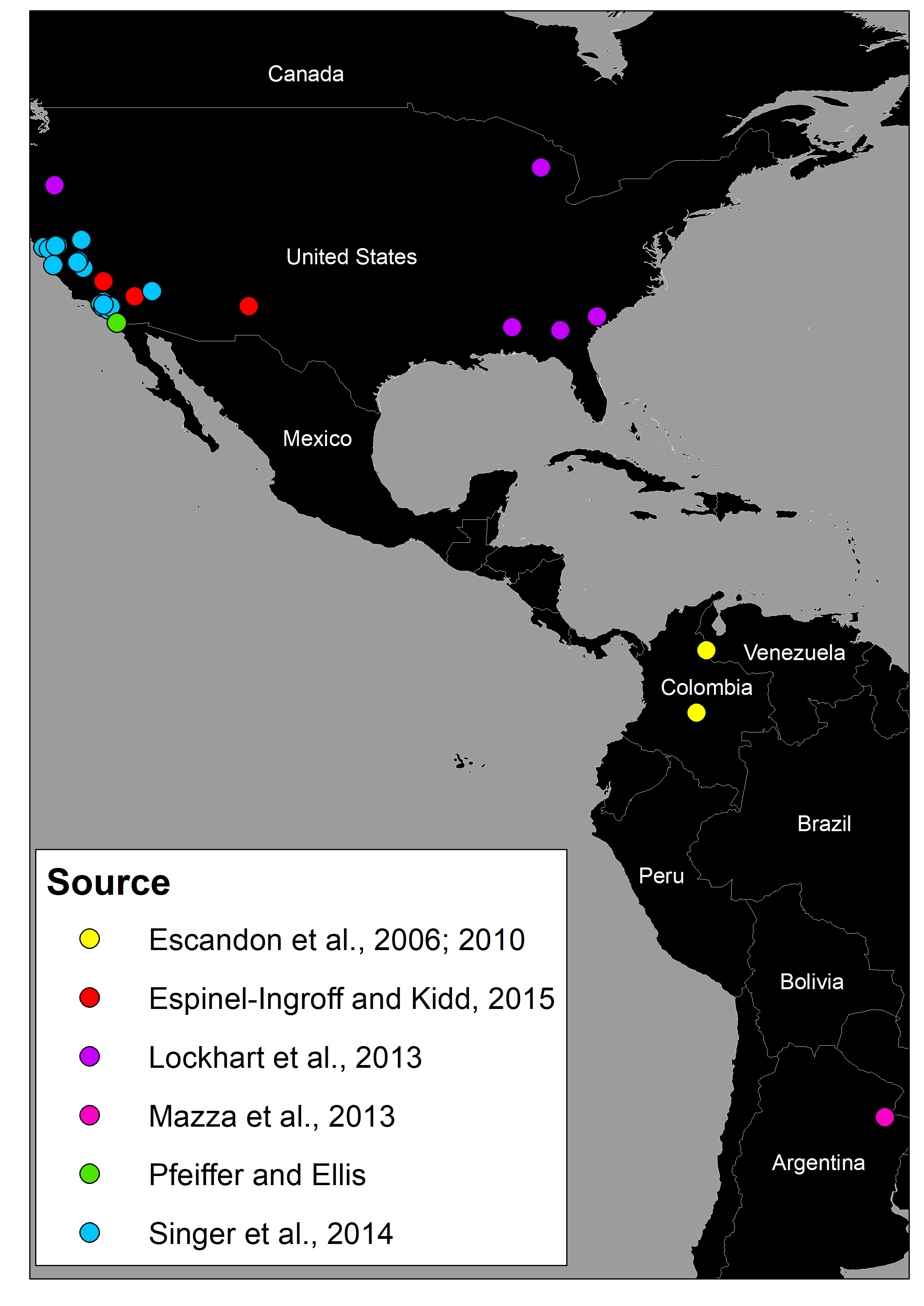


**Figure S1.** Spatial location and source of *Cryptococcus bacillisporus* occurrences used in the ENM process.

**
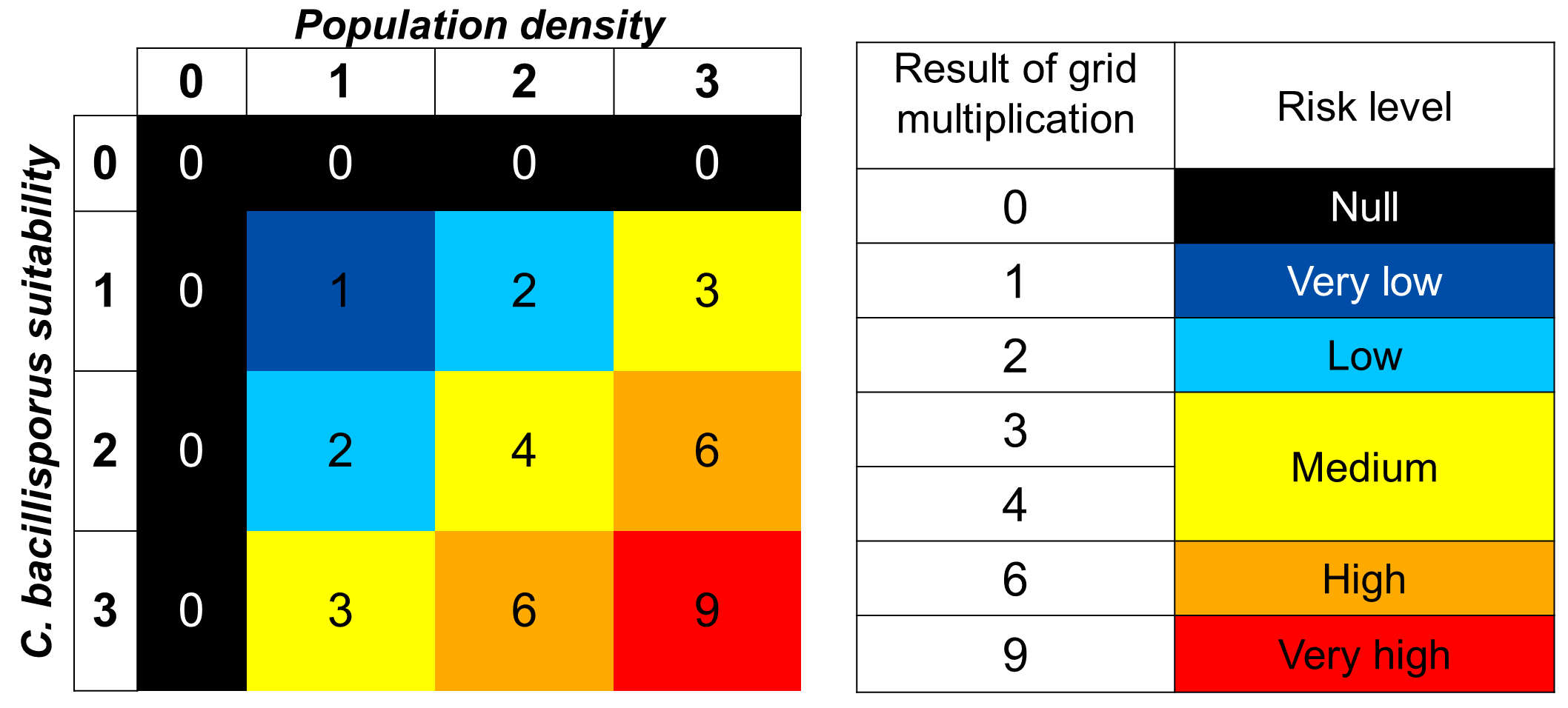
**

**Figure S2.** Double entry matrix of grid multiplication to estimate the level of exposure risk.


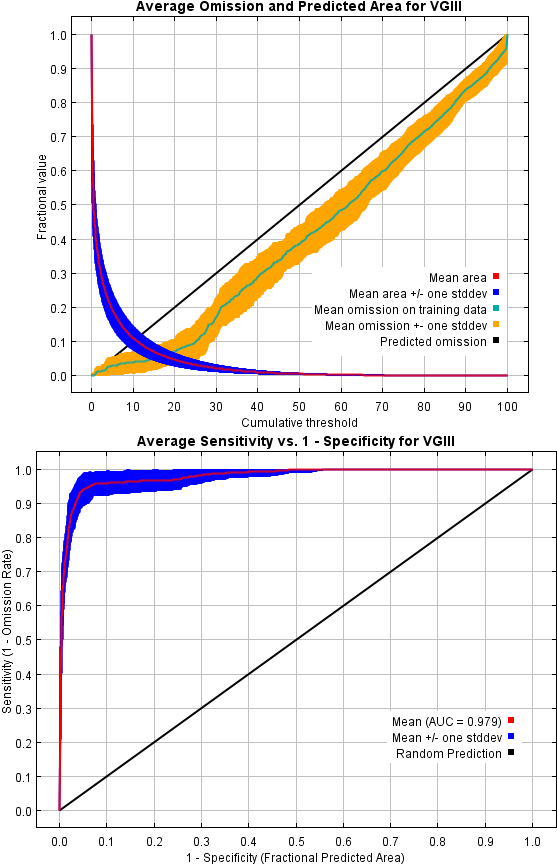


**Figure S3.** Plots of the accuracy of each model with 30-fold bootstrap technique. The upper plot corresponds to ROC curves, which represent the area under the curve (AUC). The lower plot shows the omission rate and predicted area as a function of cumulative threshold, averaged over the replicate runs (30).


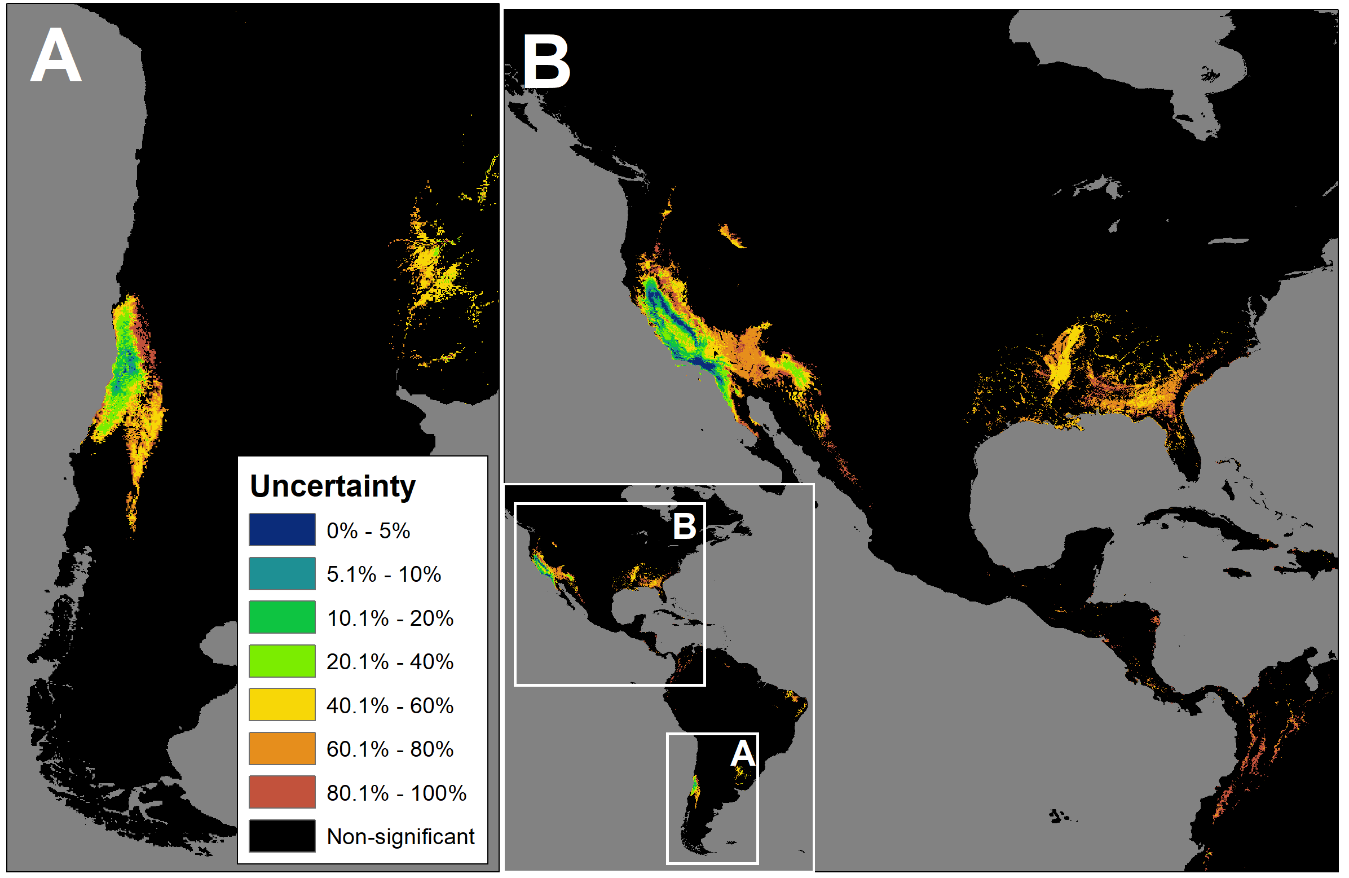


**Figure S4.** Uncertainty of the ENM prediction obtained by comparing the standard deviation of the suitability prediction with the median suitability values per pixel.


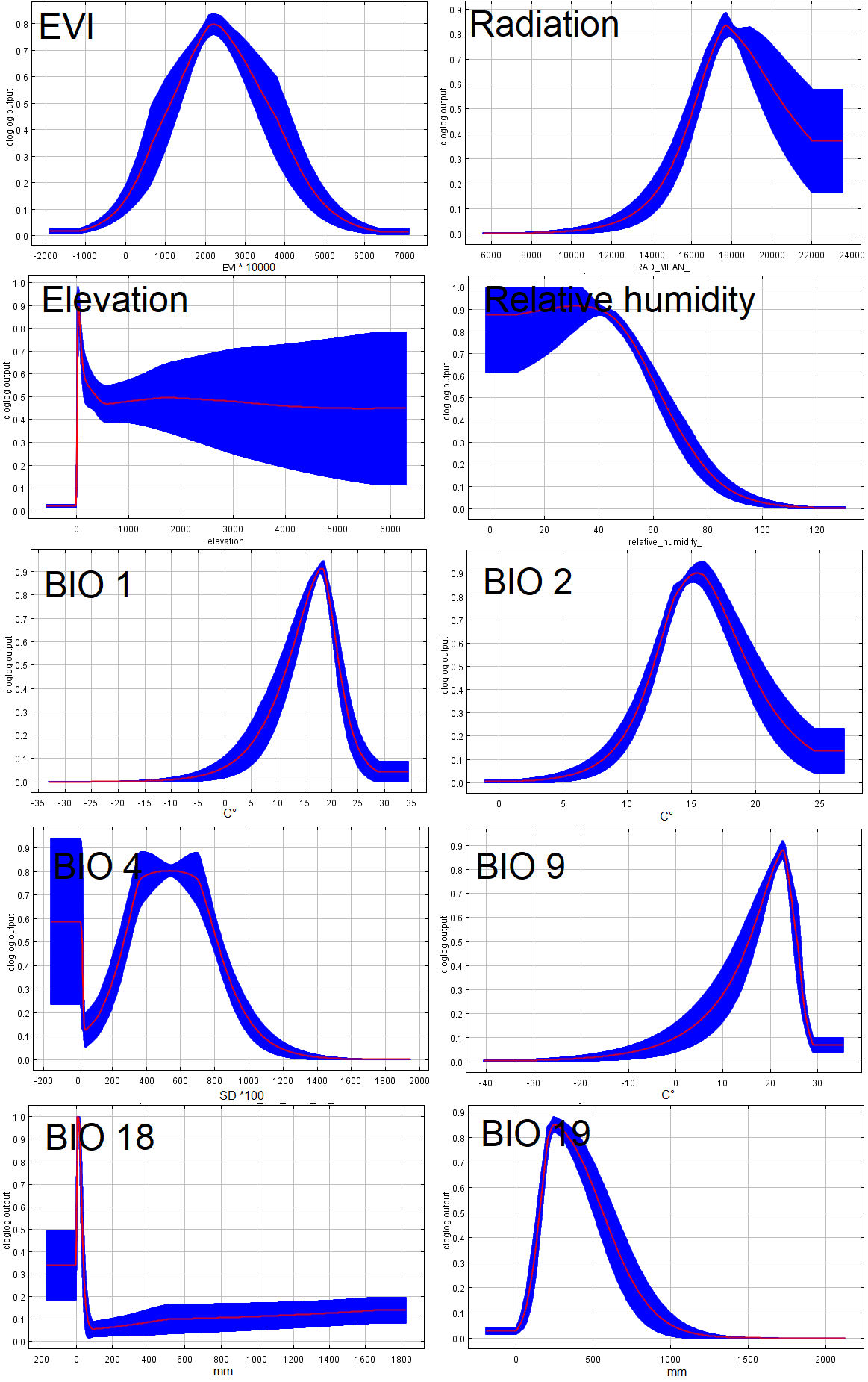


**Figure S5.** Response curves of the environmental variables in the final ENM of the *C. bacillisporus*

**Table S1.** *Cryptococcus bacillisporus* occurrences used in the ENM, showing latitude and longitude coordinates and the source of each specific data. The references are detailed below the table.

| Id | Latitude | Longitude | Source |
| --- | --- | --- | --- |
| 1 | 34.699561 | -115.811714 | Espinel-Ingroff & Kidd (2015) |
| 2 | 35.841703 | -118.191175 |  |
| 3 | 33.961929 | -107.174271 |  |
| 4 | 32.36769 | -87.210593 | Lockhart et al. (2013) |
| 5 | 32.153538 | -83.570018 |  |
| 6 | 33.200501 | -80.786048 |  |
| 7 | 44.455352 | -85.045284 |  |
| 8 | 43.122853 | -121.87934 |  |
| 9 | 35.106449 | -114.50777 | Singer et al. (2014) |
| 10 | 38.984971 | -119.84966 |  |
| 11 | 38.437695 | -122.71691 |  |
| 12 | 38.294927 | -122.312402 |  |
| 13 | 38.580462 | -121.646153 |  |
| 14 | 38.54477 | -121.812715 |  |
| 15 | 37.081402 | -121.991175 |  |
| 16 | 36.855353 | -119.694995 |  |
| 17 | 37.402629 | -120.075708 |  |
| 18 | 37.295553 | -120.147092 |  |
| 19 | 34.297433 | -118.207832 |  |
| 20 | 34.118973 | -118.207832 |  |
| 21 | 34.107076 | -118.398188 |  |
| 22 | 33.892924 | -118.207832 |  |
| 23 | 33.738259 | -117.827118 |  |
| 24 | 33.928616 | -117.636761 |  |
| 25 | 34.089707 | -118.182852 |  |
| 26 | 1.795242 | -73.213817 | Escandón et al. (2006; 2010) |
| 27 | 7.91707 | -72.508691 |  |
| 28 | 32.717518 | -117.160842 | Pfeiffer & Ellis (1991) |
| 29 | -27.462918 | -58.987296 | Mazza et al. (2013) |

**References Table S1**

Escandón, P, Sánchez, A., Firacative, C., & Castañeda, E. (2010). *Isolation of Cryptococcus gattii molecular type VGIII , from Corymbia fi cifolia detritus in Colombia*. *June*, 675–678. https://doi.org/10.3109/13693780903420633

Escandón, P, Sánchez, A., Martínez, M., Meyer, W., & Castañeda, E. (2006). Molecular epidemiology of clinical and environmental isolates of the Cryptococcus neoformans species complex reveals a high genetic diversity and the presence of the molecular type VGII mating type a in Colombia. *FEMS Yeast Research*, *6*(4), 625–635. https://doi.org/10.1111/j.1567-1364.2006.00055.x

Espinel-Ingroff, A., & Kidd, S. E. (2015). Current trends in the prevalence of Cryptococcus gattii in the United States and Canada. *Infection and Drug Resistance*, *8*, 89–97. https://doi.org/10.2147/IDR.S57686

Lockhart, S. R., Iqbal, N., Harris, J. R., Grossman, N. T., DeBess, E., Wohrle, R., Marsden-Haug, N., & Vugia, D. J. (2013). Cryptococcus gattii in the United States: Genotypic Diversity of Human and Veterinary Isolates. *PLoS ONE*, *8*(9), 27–30. https://doi.org/10.1371/journal.pone.0074737

Mazza, M., Refojo, N., Bosco-Borgeat, M. E., Taverna, C. G., Trovero, A. C., Rogé, A., & Davel, G. (2013). Cryptococcus gattii in urban trees from cities in North-eastern Argentina. *Mycoses*, *56*(6), 646–650. https://doi.org/10.1111/myc.12084

Pfeiffer, T., & Ellis, D. (1991). *Environmental Isolation of Cryptococcus neoformans gattii from California Hickman Catheter-Related Protothecal Algaemia in an Immunocompromised Child*. *163*(August 1990), 1990–1991.

Singer, L. M., Meyer, W., Firacative, C., Thompson, G. R., Samitz, E., & Sykes, J. E. (2014). Antifungal drug susceptibility and phylogenetic diversity among Cryptococcus isolates from dogs and cats in North America. *Journal of Clinical Microbiology*, *52*(6), 2061–2070. https://doi.org/10.1128/JCM.03392-13

**Table S2.** Environmental variables used in the modeling process and source of each.

| **Nomenclature** | **Variable** | **Source** |
| --- | --- | --- |
| bio1 | Annual mean temperature | Worldclim v2.0 (Fick and Hijmans, 2017) |
| bio2 | Mean temperature diurnal range (mean of monthly (max temp - min temp)) |  |
| bio3 | Isothermality (bio2/bio7) (* 100) |  |
| bio4 | Temperature seasonality (standard deviation *100) |  |
| bio5 | Max temperature of warmest month |  |
| bio6 | Min temperature of coldest month |  |
| bio7 | Temperature annual range (bio5-bio6) |  |
| bio8 | Mean temperature of wettest quarter |  |
| bio9 | Mean temperature of driest quarter |  |
| bio10 | Mean temperature of warmest quarter |  |
| bio11 | Mean temperature of coldest quarter |  |
| bio12 | Annual precipitation |  |
| bio13 | Precipitation of wettest month |  |
| bio14 | Precipitation of driest month |  |
| bio15 | Precipitation seasonality (coefficient of variation) |  |
| bio16 | Precipitation of wettest quarter |  |
| bio17 | Precipitation of driest quarter |  |
| bio18 | Precipitation of warmest quarter |  |
| bio19 | Precipitation of coldest quarter |  |
| Wind | Mean annual wind speed |  |
| SR | Annual solar radiation |  |
| RH | Relative humidity | Based on vapor preassure data from Worldclim v2.0.^1^ |
| elevation | Elevation considering meters above sea level | Shuttle Radar Topography Mission (SRTM) version 2. |
| aspect | Solar exposition of mountains in degrees from 0 to 360° |  |
| slope | Terrain slope in degrees from 0 to 90° |  |
| topodiv | Global ALOS Topographic Diversity, is a surrogate variable which represents the topographic variability of temperature and moisture. | ALOS PALSAR global radar imagery.^2^ |
| EVI | The Enhanced Vegetation Index (EVI) generated using the Near-IR, Red and Blue bands, and ranges in value from -1 to 1. Mean of all the images from 2000 to 2017. | MODIS / MCD43A4 Combined 16-Day EVI.^3^ |
| NPP | Annual Net Primary Productivity (NPP) at 500m pixel resolution. Annual NPP is derived from the sum of the 45 8-day Net Photosynthesis (PSN) products (MOD17A2H) for the given year. The PSN value is the difference of the GPP and the Maintenance Respiration (MR) (GPP-MR). | MODIS derived product, MOD17A3H.006: Terra Net Primary Production Yearly Global 500m |
| VCF | Vegetation continuous fields is a representation of the percent of canopy cover per pixel worldwide. | MODIS derived product , MOD44B.051 Terra Vegetation Continuous Fields Yearly Global 250m |
| soil_15cm | Content of soil organic carbon (SOC) in tons^-1^ per hectare from 0 to 15 cm depth. | Soil Grid 250.^4^ |
| Soil bulk | Mean soil compaction from 0 to 15 cm depth, expressed in kg/cubic meter |  |
| Soil pH | Mean soil pH * 10 in H_2_O from 0 to 15 cm depth. |  |

**References (Table S2)**

1. Fick SE, Hijmans RJ. WorldClim 2: new 1-km spatial resolution climate surfaces for global land areas. Int J Climatol 2017; 37: 4302–15.
2. Hengl T, De Jesus JM, Heuvelink GBM, et al. SoilGrids250m: Global gridded soil information based on machine learning. 2017 DOI:10.1371/journal.pone.0169748.
3. Huete A, Didan K, Miura T, Rodriguez EP, Gao X, Ferreira LG. Overview of the radiometric and biophysical performance of the MODIS vegetation indices. Remote Sens Environ 2002; 83: 195–213.
4. Theobald DM, Harrison-Atlas D, Monahan WB, Albano CM. Ecologically-Relevant Maps of Landforms and Physiographic Diversity for Climate Adaptation Planning. PLoS One 2015; 10: e0143619.

**Table S3.** Percent contribution and permutation importance of the environmental variables in the ENM of *Cryptococcus bacillisporus*.

| Variable | Percent contribution | Permutation importance |
| --- | --- | --- |
| BIO 18 | 51.2 | 2.1 |
| BIO 19 | 22.1 | 51.4 |
| SR | 10.2 | 18.2 |
| EVI | 4.4 | 8.9 |
| BIO 9 | 2.8 | 2.4 |
| BIO 4 | 2.7 | 2.5 |
| BIO 1 | 1.9 | 3.6 |
| RH | 1.9 | 6 |
| ELEVATION | 1.7 | 3.1 |
| BIO 2 | 0.9 | 1 |

**Table S4.** People per province and level of risk in Chile.

| Province | Very low | Low | Medium | High | Very high | Total at risk |
| --- | --- | --- | --- | --- | --- | --- |
| Santiago | 0 | 77 | 39181 | 164609 | 5041621 | 5245487 |
| Cordillera | 5170 | 4405 | 12848 | 47352 | 996254 | 1066029 |
| Maipo | 0 | 0 | 4616 | 42827 | 681246 | 728689 |
| Cachapoal | 79 | 32865 | 134765 | 430443 | 63049 | 661200 |
| Valparaíso | 0 | 0 | 7121 | 158269 | 461652 | 627042 |
| Talagante | 0 | 0 | 0 | 16642 | 525534 | 542175 |
| Marga marga | 0 | 0 | 0 | 15462 | 401765 | 417228 |
| Chacabuco | 0 | 758 | 4637 | 37401 | 320242 | 363038 |
| Talca | 29060 | 69600 | 35083 | 173207 | 30251 | 337201 |
| Melipilla | 0 | 652 | 19309 | 140458 | 127420 | 287839 |
| Curicó | 848 | 75812 | 144644 | 33856 | 4339 | 259499 |
| Colchagua | 0 | 46183 | 78388 | 55241 | 22151 | 201964 |
| Quillota | 0 | 0 | 18 | 48353 | 146720 | 195091 |
| San Antonio | 0 | 3 | 1164 | 20789 | 164237 | 186192 |
| San Felipe de Aconcagua | 193 | 8197 | 4490 | 51290 | 103707 | 167877 |
| Linares | 12149 | 94961 | 44333 | 808 | 0 | 152251 |
| Limarí | 15401 | 90829 | 44482 | 1 | 0 | 150714 |
| Ñuble | 564 | 54952 | 77950 | 0 | 0 | 133465 |
| Elqui | 6578 | 6770 | 115548 | 0 | 0 | 128896 |
| Los Andes | 0 | 43397 | 15522 | 34580 | 10679 | 104178 |
| Concepción | 0 | 0 | 91080 | 0 | 0 | 91080 |
| Petorca | 714 | 5125 | 14714 | 64160 | 564 | 85277 |
| Choapa | 10710 | 15527 | 22709 | 24439 | 0 | 73385 |
| Cardenal Caro | 165 | 8588 | 20325 | 17163 | 0 | 46241 |
| Cauquenes | 11 | 25679 | 18710 | 0 | 0 | 44399 |
| Biobío | 0 | 2113 | 0 | 0 | 0 | 2113 |

**Table S5.** People per county and level of risk in the USA.

| County | Very low | Low | Medium | High | Very high | Total at risk |
| --- | --- | --- | --- | --- | --- | --- |
| Los Angeles | 1401 | 2986 | 9601 | 161820 | 8767113 | 8942921 |
| Maricopa | 3759 | 32988 | 3888316 | 43818 | 0 | 3968881 |
| Orange | 56 | 4068 | 525588 | 19539 | 2638390 | 3187641 |
| San Diego | 1161 | 5842 | 7782 | 390530 | 2671542 | 3076857 |
| Clark | 3477 | 15245 | 2631211 | 0 | 0 | 2649932 |
| Riverside | 1684 | 24439 | 421627 | 345038 | 1697403 | 2490191 |
| San Bernardino | 5210 | 24255 | 246877 | 254139 | 1695924 | 2226405 |
| Santa Clara | 409 | 3685 | 5406 | 42089 | 1576749 | 1628338 |
| Sacramento | 27 | 176 | 2766 | 17805 | 1529997 | 1550771 |
| Alameda | 0 | 292 | 3643 | 18368 | 1348268 | 1370572 |
| Harris | 263 | 7227 | 1161428 | 0 | 0 | 1168919 |
| Contra Costa | 8 | 288 | 6809 | 33885 | 1017376 | 1058366 |
| Fresno | 998 | 8941 | 46105 | 89778 | 875352 | 1021173 |
| Kern | 2852 | 25947 | 177553 | 673244 | 26485 | 906081 |
| Shelby | 1143 | 29370 | 802731 | 0 | 0 | 833244 |
| San Mateo | 441 | 4689 | 15281 | 74005 | 566181 | 660597 |
| Pima | 2011 | 12270 | 628683 | 0 | 0 | 642964 |
| San Joaquin | 905 | 5001 | 20352 | 98264 | 509854 | 634377 |
| Ventura | 25 | 0 | 2406 | 22972 | 606509 | 631912 |
| Jefferson | 6140 | 35912 | 556045 | 0 | 0 | 598096 |
| Stanislaus | 242 | 8838 | 11008 | 135112 | 396988 | 552187 |
| Tulare | 894 | 4974 | 20712 | 77025 | 416825 | 520429 |
| Sonoma | 1942 | 11802 | 34430 | 146755 | 261698 | 456626 |
| Placer | 1047 | 8837 | 10330 | 39219 | 389181 | 448613 |
| Fulton | 151 | 584 | 436055 | 0 | 0 | 436790 |
| San Francisco | 0 | 9 | 22115 | 112814 | 281848 | 416786 |
| Dallas | 2875 | 10963 | 401980 | 0 | 0 | 415818 |
| Gwinnett | 0 | 0 | 414582 | 0 | 0 | 414582 |
| Montgomery | 4678 | 17466 | 382477 | 0 | 0 | 404622 |
| Solano | 1 | 446 | 3503 | 13329 | 386154 | 403433 |
| Hillsborough | 62 | 0 | 397606 | 0 | 0 | 397669 |
| Santa Barbara | 0 | 75 | 5147 | 10382 | 351260 | 366864 |
| Monterey | 635 | 2795 | 27657 | 66375 | 266885 | 364347 |
| Pinal | 2573 | 16296 | 287790 | 13125 | 4490 | 324273 |
| Lake | 1379 | 13017 | 244230 | 20791 | 34594 | 314010 |
| Merced | 222 | 8409 | 15197 | 101206 | 184988 | 310022 |
| Richland | 1327 | 7647 | 285955 | 0 | 0 | 294929 |
| Madison | 4130 | 29329 | 233223 | 0 | 0 | 266682 |
| Santa Cruz | 1148 | 12160 | 40029 | 73104 | 135617 | 262058 |
| Cumberland | 164 | 3915 | 242726 | 0 | 0 | 246805 |
| Yolo | 0 | 103 | 4078 | 8446 | 229480 | 242107 |
| Jackson | 10075 | 62593 | 161336 | 1908 | 0 | 235912 |
| Pinellas | 0 | 0 | 234361 | 0 | 0 | 234361 |
| Houston | 2571 | 34708 | 192022 | 0 | 0 | 229301 |
| Marin | 1219 | 5566 | 12677 | 83547 | 125845 | 228854 |
| Polk | 173 | 3699 | 213826 | 0 | 0 | 217698 |
| Mohave | 5515 | 28415 | 148559 | 34272 | 0 | 216761 |
| Davidson | 0 | 953 | 209567 | 0 | 0 | 210520 |
| San Luis Obispo | 0 | 46 | 8107 | 30526 | 169885 | 208563 |
| Leon | 1049 | 11118 | 196295 | 0 | 0 | 208462 |
| Cobb | 0 | 0 | 204480 | 0 | 0 | 204480 |
| Pasco | 0 | 4 | 197719 | 0 | 0 | 197722 |
| El Dorado | 1182 | 7946 | 23494 | 73240 | 90134 | 195996 |
| Escambia | 5928 | 26061 | 163183 | 0 | 0 | 195172 |
| Butte | 1072 | 3569 | 18125 | 38717 | 132186 | 193669 |
| Duval | 265 | 5687 | 183545 | 0 | 0 | 189496 |
| Lexington | 15 | 13064 | 176234 | 0 | 0 | 189314 |
| Washington | 12162 | 37218 | 138622 | 0 | 0 | 188002 |
| Lee | 5902 | 40382 | 139892 | 0 | 0 | 186176 |
| Orleans | 68 | 2040 | 182313 | 0 | 0 | 184421 |
| Madera | 485 | 1803 | 7346 | 34516 | 140000 | 184150 |
| Tuscaloosa | 1562 | 23662 | 148554 | 0 | 0 | 173778 |
| San Juan | 0 | 0 | 170747 | 0 | 0 | 170747 |
| Mecklenburg | 513 | 1066 | 169041 | 0 | 0 | 170620 |
| Kings | 0 | 2223 | 15397 | 71518 | 77535 | 166673 |
| Richmond | 505 | 9967 | 136568 | 14432 | 0 | 161472 |
| Bibb | 1968 | 9894 | 149529 | 0 | 0 | 161392 |
| DeKalb | 153 | 141 | 158217 | 0 | 0 | 158511 |
| Washoe | 1806 | 10121 | 131846 | 9192 | 3142 | 156108 |
| Clayton | 0 | 0 | 153383 | 0 | 0 | 153383 |
| Columbia | 1007 | 11594 | 131098 | 0 | 0 | 143699 |
| Charleston | 159 | 7245 | 134038 | 0 | 0 | 141442 |
| Tarrant | 0 | 0 | 139902 | 0 | 0 | 139902 |
| Aiken | 1277 | 28623 | 105786 | 2908 | 0 | 138594 |
| Yavapai | 4074 | 20806 | 110545 | 2561 | 0 | 137987 |
| Mobile | 1897 | 13918 | 121303 | 0 | 0 | 137118 |
| Muscogee | 474 | 4293 | 132226 | 0 | 0 | 136994 |
| Hamilton | 1339 | 4038 | 129792 | 0 | 0 | 135169 |
| Wake | 5 | 189 | 133597 | 0 | 0 | 133791 |
| Lowndes | 4910 | 25404 | 102278 | 0 | 0 | 132591 |
| Caddo | 825 | 1727 | 124568 | 0 | 0 | 127120 |
| Shasta | 3950 | 7901 | 10347 | 22425 | 77772 | 122394 |
| Sutter | 0 | 0 | 2320 | 11248 | 107878 | 121446 |
| Napa | 502 | 1335 | 6372 | 12102 | 100442 | 120753 |
| Clay | 5673 | 15591 | 99242 | 0 | 0 | 120507 |
| Hinds | 274 | 1857 | 115292 | 0 | 0 | 117423 |
| Sumter | 5989 | 14615 | 94816 | 0 | 0 | 115420 |
| Henry | 3255 | 10079 | 100316 | 0 | 0 | 113650 |
| Okaloosa | 2106 | 13517 | 97749 | 0 | 0 | 113372 |
| Bay | 1268 | 8661 | 98703 | 0 | 0 | 108632 |
| Santa Rosa | 2963 | 19722 | 84581 | 0 | 0 | 107266 |
| Rutherford | 0 | 1755 | 101807 | 0 | 0 | 103562 |
| Nevada | 1017 | 9067 | 35621 | 42874 | 14770 | 103349 |
| Pulaski | 2251 | 8621 | 87751 | 0 | 0 | 98623 |
| Fort Bend | 39 | 2849 | 91277 | 0 | 0 | 94166 |
| Dougherty | 541 | 9124 | 84153 | 0 | 0 | 93819 |
| Rankin | 326 | 7962 | 78281 | 3557 | 0 | 90126 |
| Chatham | 298 | 4683 | 84536 | 0 | 0 | 89517 |
| Cherokee | 694 | 7990 | 80556 | 0 | 0 | 89240 |
| Yuba | 748 | 1763 | 2567 | 15988 | 67465 | 88531 |
| Collin | 0 | 5448 | 82750 | 0 | 0 | 88198 |
| Beaufort | 522 | 6815 | 79163 | 0 | 0 | 86501 |
| Lauderdale | 2141 | 13783 | 69973 | 0 | 0 | 85897 |
| Clarke | 3022 | 9583 | 72578 | 0 | 0 | 85183 |
| Baldwin | 2320 | 24776 | 55762 | 2120 | 0 | 84978 |
| McLennan | 829 | 5566 | 77025 | 0 | 0 | 83420 |
| Coweta | 0 | 4472 | 75708 | 0 | 0 | 80181 |
| East Baton Rouge | 302 | 1354 | 78240 | 0 | 0 | 79896 |
| Rockwall | 0 | 740 | 77020 | 0 | 0 | 77760 |
| Denton | 23 | 1847 | 73901 | 0 | 0 | 75772 |
| Marion | 2432 | 18760 | 53888 | 0 | 0 | 75081 |
| Coffee | 6055 | 30865 | 37947 | 0 | 0 | 74868 |
| DeSoto | 618 | 3185 | 69493 | 0 | 0 | 73295 |
| Fayette | 308 | 6982 | 60246 | 0 | 0 | 67535 |
| Palm Beach | 0 | 0 | 64780 | 0 | 0 | 64780 |
| Henderson | 664 | 5327 | 58529 | 0 | 0 | 64520 |
| Tehama | 189 | 547 | 4227 | 24259 | 35078 | 64299 |
| Mendocino | 2894 | 12701 | 18544 | 25216 | 0 | 59355 |
| Morgan | 67 | 4138 | 54184 | 0 | 0 | 58389 |
| Bulloch | 1104 | 15912 | 40472 | 0 | 0 | 57488 |
| San Benito | 0 | 42 | 1616 | 8552 | 45994 | 56205 |
| Manatee | 98 | 1053 | 54486 | 0 | 0 | 55637 |
| Tuolumne | 316 | 5013 | 3219 | 22471 | 24527 | 55546 |
| Williamson | 1807 | 5733 | 47528 | 0 | 0 | 55068 |
| Elmore | 1416 | 21304 | 30054 | 0 | 0 | 52774 |
| Monroe | 6758 | 21477 | 24293 | 0 | 0 | 52528 |
| Newton | 83 | 1046 | 50949 | 0 | 0 | 52078 |
| Etowah | 23 | 2309 | 49681 | 0 | 0 | 52013 |
| Vanderburgh | 130 | 1717 | 49967 | 0 | 0 | 51814 |
| Crittenden | 3208 | 7132 | 40610 | 0 | 0 | 50950 |
| Greenville | 0 | 0 | 50862 | 0 | 0 | 50862 |
| Jones | 1158 | 14127 | 35522 | 0 | 0 | 50807 |
| Volusia | 113 | 1634 | 49001 | 0 | 0 | 50749 |
| Autauga | 1194 | 12160 | 37095 | 0 | 0 | 50449 |
| New Hanover | 0 | 0 | 50043 | 0 | 0 | 50043 |
| Knox | 0 | 0 | 48650 | 0 | 0 | 48650 |
| Floyd | 342 | 2800 | 44189 | 0 | 0 | 47331 |
| Douglas | 379 | 2232 | 41041 | 3508 | 0 | 47161 |
| Anderson | 127 | 4951 | 42013 | 0 | 0 | 47092 |
| Calaveras | 470 | 5839 | 10153 | 17857 | 12685 | 47003 |
| Yuma | 803 | 4345 | 40943 | 0 | 0 | 46091 |
| Calhoun | 6021 | 22228 | 16523 | 0 | 0 | 44772 |
| Citrus | 134 | 6194 | 38126 | 0 | 0 | 44454 |
| Rockdale | 0 | 0 | 44438 | 0 | 0 | 44438 |
| Union | 3954 | 8000 | 31864 | 0 | 0 | 43818 |
| Mississippi | 4405 | 15582 | 23030 | 0 | 0 | 43017 |
| Russell | 2290 | 12802 | 27762 | 0 | 0 | 42854 |
| Troup | 558 | 10997 | 31175 | 0 | 0 | 42731 |
| York | 0 | 0 | 42470 | 0 | 0 | 42470 |
| Bell | 1038 | 7732 | 33469 | 0 | 0 | 42239 |
| Gila | 1980 | 6130 | 20913 | 13173 | 0 | 42196 |
| Forrest | 413 | 4421 | 36741 | 0 | 0 | 41575 |
| Hall | 0 | 65 | 40930 | 0 | 0 | 40995 |
| Greene | 7212 | 13610 | 20089 | 0 | 0 | 40910 |
| Forsyth | 0 | 240 | 39862 | 0 | 0 | 40102 |
| St. Johns | 0 | 1072 | 38673 | 0 | 0 | 39745 |
| Lamar | 212 | 2056 | 37132 | 0 | 0 | 39399 |
| Tift | 624 | 11727 | 26384 | 0 | 0 | 38734 |
| Garland | 0 | 1284 | 37187 | 0 | 0 | 38471 |
| Carolina | 0 | 0 | 38121 | 0 | 0 | 38121 |
| Amador | 128 | 6232 | 4971 | 9426 | 17041 | 37798 |
| Lincoln | 1772 | 3721 | 32078 | 0 | 0 | 37571 |
| Spartanburg | 0 | 1290 | 36254 | 0 | 0 | 37544 |
| Colquitt | 2253 | 20597 | 14138 | 0 | 0 | 36988 |
| Bossier | 514 | 1784 | 34143 | 0 | 0 | 36440 |
| Walton | 3109 | 14930 | 18155 | 0 | 0 | 36195 |
| Dale | 373 | 11836 | 23906 | 0 | 0 | 36115 |
| Hernando | 166 | 1159 | 34762 | 0 | 0 | 36087 |
| Nye | 775 | 8806 | 26298 | 0 | 0 | 35879 |
| Thomas | 2875 | 17789 | 13994 | 0 | 0 | 34658 |
| Sumner | 0 | 836 | 33671 | 0 | 0 | 34506 |
| Glynn | 188 | 7509 | 26585 | 0 | 0 | 34282 |
| Peach | 101 | 4369 | 29443 | 0 | 0 | 33913 |
| Seminole | 2212 | 4950 | 26027 | 360 | 0 | 33550 |
| St. Clair | 10 | 8354 | 24647 | 0 | 0 | 33011 |
| Laurens | 3176 | 19952 | 9691 | 0 | 0 | 32819 |
| Lassen | 1880 | 7481 | 12750 | 10477 | 0 | 32587 |
| Wilson | 0 | 4177 | 28289 | 0 | 0 | 32466 |
| Josephine | 0 | 799 | 31617 | 0 | 0 | 32416 |
| Orangeburg | 578 | 8392 | 23063 | 0 | 0 | 32034 |
| Siskiyou | 4686 | 14851 | 12149 | 0 | 0 | 31686 |
| Calcasieu | 145 | 153 | 31380 | 0 | 0 | 31678 |
| Harrison | 230 | 2528 | 28857 | 0 | 0 | 31614 |
| Butler | 4302 | 7244 | 19410 | 0 | 0 | 30956 |
| St. Tammany | 0 | 3091 | 27237 | 0 | 0 | 30328 |
| Colbert | 249 | 7327 | 22643 | 0 | 0 | 30219 |
| Ware | 2687 | 8268 | 18887 | 0 | 0 | 29842 |
| Glenn | 0 | 0 | 3163 | 5557 | 20403 | 29123 |
| Talladega | 384 | 19050 | 9659 | 0 | 0 | 29093 |
| Covington | 4235 | 12911 | 11581 | 0 | 0 | 28727 |
| Oktibbeha | 448 | 4190 | 23841 | 0 | 0 | 28478 |
| Sebastian | 0 | 578 | 27719 | 0 | 0 | 28297 |
| Darlington | 302 | 13950 | 13570 | 0 | 0 | 27822 |
| Alachua | 239 | 6275 | 20324 | 0 | 0 | 26838 |
| Toombs | 1324 | 12585 | 12743 | 0 | 0 | 26653 |
| Carroll | 844 | 1316 | 24459 | 0 | 0 | 26619 |
| Robeson | 0 | 7942 | 18544 | 0 | 0 | 26485 |
| Decatur | 4557 | 11831 | 9083 | 0 | 0 | 25470 |
| Sunflower | 2259 | 7589 | 12364 | 3142 | 0 | 25353 |
| Geneva | 3459 | 13937 | 7924 | 0 | 0 | 25321 |
| Ellis | 277 | 4299 | 20683 | 0 | 0 | 25260 |
| Webster | 2076 | 12251 | 10583 | 0 | 0 | 24910 |
| Grady | 1992 | 10456 | 11851 | 0 | 0 | 24299 |
| Macon | 4647 | 8395 | 11139 | 0 | 0 | 24182 |
| Whitfield | 0 | 0 | 23887 | 0 | 0 | 23887 |
| Dunklin | 2932 | 9389 | 11207 | 0 | 0 | 23528 |
| Tattnall | 2158 | 9564 | 11670 | 0 | 0 | 23392 |
| Colusa | 0 | 0 | 2700 | 3018 | 17538 | 23256 |
| Bartow | 0 | 3717 | 19087 | 0 | 0 | 22804 |
| Gregg | 0 | 1502 | 21213 | 0 | 0 | 22715 |
| Tipton | 520 | 12303 | 9617 | 0 | 0 | 22440 |
| Craighead | 3052 | 8783 | 10579 | 0 | 0 | 22414 |
| Paulding | 0 | 0 | 21762 | 0 | 0 | 21762 |
| West Baton Rouge | 77 | 685 | 20837 | 0 | 0 | 21599 |
| Kershaw | 769 | 8953 | 11733 | 0 | 0 | 21455 |
| Mitchell | 4789 | 6123 | 10512 | 0 | 0 | 21423 |
| Coahoma | 1482 | 5472 | 14400 | 0 | 0 | 21353 |
| Limestone | 701 | 7619 | 12632 | 0 | 0 | 20953 |
| Barbour | 3029 | 9253 | 8625 | 0 | 0 | 20907 |
| Chambers | 526 | 2838 | 17508 | 0 | 0 | 20872 |
| Oconee | 17 | 5533 | 15249 | 0 | 0 | 20800 |
| Pike | 936 | 2114 | 17627 | 0 | 0 | 20677 |
| Walker | 328 | 15741 | 4475 | 0 | 0 | 20544 |
| Yazoo | 2103 | 3109 | 15300 | 0 | 0 | 20512 |
| Putnam | 477 | 14529 | 5357 | 0 | 0 | 20363 |
| Leflore | 1194 | 7084 | 11657 | 0 | 0 | 19935 |
| Berkeley | 113 | 8682 | 11130 | 0 | 0 | 19926 |
| Lonoke | 3098 | 7190 | 9611 | 0 | 0 | 19899 |
| Telfair | 3323 | 7173 | 9017 | 0 | 0 | 19513 |
| Emanuel | 5048 | 6784 | 7554 | 0 | 0 | 19386 |
| Guaynabo | 0 | 0 | 19306 | 0 | 0 | 19306 |
| New Madrid | 2536 | 7072 | 9680 | 0 | 0 | 19288 |
| Sarasota | 0 | 0 | 19022 | 0 | 0 | 19022 |
| Lawrence | 2220 | 9933 | 6595 | 0 | 0 | 18749 |
| Crawford | 1078 | 9470 | 7824 | 0 | 0 | 18372 |
| Scott | 1774 | 6339 | 9850 | 0 | 0 | 17963 |
| Appling | 3466 | 9909 | 4365 | 0 | 0 | 17740 |
| Scotland | 218 | 4392 | 12967 | 0 | 0 | 17578 |
| Poinsett | 2310 | 8891 | 6197 | 0 | 0 | 17398 |
| Caguas | 0 | 0 | 17327 | 0 | 0 | 17327 |
| Hot Spring | 587 | 5273 | 11386 | 0 | 0 | 17246 |
| Worth | 3843 | 10573 | 2781 | 0 | 0 | 17197 |
| Carson City | 0 | 1708 | 15484 | 0 | 0 | 17192 |
| Hoke | 0 | 2051 | 15096 | 0 | 0 | 17147 |
| Barnwell | 1560 | 11422 | 4140 | 0 | 0 | 17122 |
| Plumas | 1623 | 6073 | 2504 | 6604 | 0 | 16804 |
| Iredell | 0 | 356 | 16434 | 0 | 0 | 16789 |
| Brevard | 0 | 0 | 16546 | 0 | 0 | 16546 |
| Baker | 2941 | 5692 | 7704 | 0 | 0 | 16336 |
| Mariposa | 255 | 836 | 3985 | 11188 | 0 | 16264 |
| Jeff Davis | 2105 | 7226 | 6865 | 0 | 0 | 16196 |
| Pemiscot | 3195 | 6998 | 5973 | 0 | 0 | 16167 |
| Pickens | 925 | 4046 | 10781 | 0 | 0 | 15752 |
| Hunt | 0 | 3466 | 12178 | 0 | 0 | 15644 |
| Klickitat | 2438 | 3691 | 7721 | 1631 | 0 | 15481 |
| Wayne | 3575 | 7798 | 3944 | 0 | 0 | 15317 |
| Nassau | 0 | 2552 | 12739 | 0 | 0 | 15291 |
| Dodge | 3608 | 9420 | 2192 | 0 | 0 | 15221 |
| Holmes | 2212 | 6247 | 6696 | 0 | 0 | 15155 |
| Dooly | 2928 | 5315 | 6743 | 0 | 0 | 14986 |
| Gadsden | 885 | 8669 | 5332 | 0 | 0 | 14887 |
| Wasco | 774 | 2862 | 1733 | 9504 | 0 | 14873 |
| Trinity | 2268 | 6101 | 6087 | 0 | 0 | 14456 |
| Cook | 656 | 7026 | 6727 | 0 | 0 | 14410 |
| McCracken | 0 | 2526 | 11823 | 0 | 0 | 14349 |
| Edgefield | 71 | 5813 | 8218 | 0 | 0 | 14102 |
| Brunswick | 18 | 119 | 13767 | 0 | 0 | 13904 |
| Stoddard | 3644 | 6572 | 3411 | 0 | 0 | 13628 |
| Bleckley | 1165 | 6110 | 6294 | 0 | 0 | 13569 |
| Levy | 359 | 13060 | 0 | 0 | 0 | 13419 |
| Crisp | 1726 | 5743 | 5891 | 0 | 0 | 13360 |
| Harnett | 0 | 4316 | 8914 | 0 | 0 | 13230 |
| Bolivar | 1363 | 6587 | 5153 | 0 | 0 | 13103 |
| Ouachita | 1535 | 4322 | 7180 | 0 | 0 | 13037 |
| Wheeler | 2207 | 2881 | 7907 | 0 | 0 | 12995 |
| Burke | 4307 | 8305 | 0 | 0 | 0 | 12612 |
| Mono | 700 | 2163 | 1218 | 8529 | 0 | 12610 |
| Ben Hill | 1757 | 2010 | 8802 | 0 | 0 | 12568 |
| Spalding | 0 | 136 | 12392 | 0 | 0 | 12529 |
| Dyer | 1946 | 7431 | 2912 | 0 | 0 | 12288 |
| Phillips | 2104 | 3912 | 6227 | 0 | 0 | 12243 |
| Warren | 693 | 2620 | 8907 | 0 | 0 | 12220 |
| Arkansas | 1798 | 2933 | 7382 | 0 | 0 | 12113 |
| Johnson | 2033 | 6672 | 3407 | 0 | 0 | 12112 |
| Berrien | 2619 | 7276 | 2176 | 0 | 0 | 12070 |
| Tallahatchie | 2285 | 3035 | 6747 | 0 | 0 | 12067 |
| Inyo | 1249 | 2288 | 8479 | 0 | 0 | 12016 |
| Ada | 361 | 2923 | 8626 | 0 | 0 | 11910 |
| Marlboro | 671 | 7726 | 3469 | 0 | 0 | 11866 |
| Hood River | 8 | 399 | 11395 | 0 | 0 | 11802 |
| Florence | 0 | 667 | 11052 | 0 | 0 | 11719 |
| Bacon | 2246 | 7668 | 1793 | 0 | 0 | 11707 |
| Tunica | 2067 | 6649 | 2948 | 0 | 0 | 11663 |
| Marshall | 662 | 6650 | 4311 | 0 | 0 | 11624 |
| St. Bernard | 88 | 0 | 11252 | 0 | 0 | 11340 |
| Brooks | 2400 | 5238 | 3696 | 0 | 0 | 11334 |
| Jasper | 505 | 1626 | 8994 | 0 | 0 | 11125 |
| Kaufman | 0 | 3687 | 7293 | 0 | 0 | 10980 |
| Perry | 3592 | 5398 | 1988 | 0 | 0 | 10979 |
| Osceola | 96 | 327 | 10267 | 0 | 0 | 10690 |
| Ashley | 1622 | 4502 | 4472 | 0 | 0 | 10596 |
| Faulkner | 86 | 2212 | 8239 | 0 | 0 | 10538 |
| Alcorn | 14 | 2234 | 8167 | 0 | 0 | 10415 |
| Desha | 992 | 3679 | 5701 | 0 | 0 | 10371 |
| Chicot | 1094 | 4700 | 4554 | 0 | 0 | 10348 |
| Obion | 1030 | 6878 | 2327 | 0 | 0 | 10235 |
| St. Francis | 2481 | 4029 | 3715 | 0 | 0 | 10224 |
| Suwannee | 176 | 5363 | 4644 | 0 | 0 | 10182 |
| Hardin | 504 | 3219 | 6073 | 0 | 0 | 9796 |
| Drew | 1519 | 934 | 7326 | 0 | 0 | 9779 |
| Tallapoosa | 1280 | 7309 | 1089 | 0 | 0 | 9678 |
| Lanier | 919 | 3369 | 5251 | 0 | 0 | 9539 |
| Crockett | 1826 | 3699 | 3908 | 0 | 0 | 9433 |
| La Paz | 1594 | 7496 | 116 | 162 | 0 | 9368 |
| Chilton | 1273 | 8075 | 0 | 0 | 0 | 9348 |
| Livingston | 342 | 4252 | 4313 | 0 | 0 | 8907 |
| Travis | 272 | 2193 | 6386 | 0 | 0 | 8851 |
| Terrell | 1881 | 2004 | 4953 | 0 | 0 | 8838 |
| Randolph | 2472 | 6339 | 0 | 0 | 0 | 8812 |
| Posey | 424 | 2368 | 5964 | 0 | 0 | 8756 |
| Greenwood | 0 | 1945 | 6809 | 0 | 0 | 8754 |
| Pope | 299 | 1746 | 6538 | 0 | 0 | 8583 |
| Bradford | 491 | 5925 | 2126 | 0 | 0 | 8542 |
| Wilcox | 4178 | 4333 | 0 | 0 | 0 | 8512 |
| Early | 3403 | 2880 | 2163 | 0 | 0 | 8446 |
| Vernon | 188 | 501 | 7615 | 0 | 0 | 8304 |
| Arecibo | 0 | 0 | 8297 | 0 | 0 | 8297 |
| Georgetown | 18 | 148 | 8077 | 0 | 0 | 8243 |
| Massac | 169 | 1364 | 6616 | 0 | 0 | 8149 |
| Highlands | 0 | 0 | 8123 | 0 | 0 | 8123 |
| Bradley | 1536 | 2491 | 4074 | 0 | 0 | 8101 |
| Sabine | 774 | 5132 | 1674 | 396 | 0 | 7976 |
| Guadalupe | 0 | 1579 | 6371 | 0 | 0 | 7950 |
| Humphreys | 2068 | 1966 | 3641 | 0 | 0 | 7676 |
| Quitman | 1698 | 4119 | 1836 | 0 | 0 | 7653 |
| Allendale | 1477 | 3069 | 3097 | 0 | 0 | 7643 |
| West Carroll | 2215 | 3385 | 1973 | 0 | 0 | 7573 |
| Acadia | 1137 | 6405 | 0 | 0 | 0 | 7542 |
| Lancaster | 0 | 215 | 7315 | 0 | 0 | 7530 |
| Irwin | 2867 | 4555 | 99 | 0 | 0 | 7521 |
| Hale | 2280 | 5220 | 3 | 0 | 0 | 7503 |
| Stewart | 1708 | 3267 | 2524 | 0 | 0 | 7500 |
| Panola | 1422 | 3113 | 2908 | 0 | 0 | 7443 |
| Avoyelles | 384 | 2793 | 4248 | 0 | 0 | 7425 |
| Atkinson | 1546 | 3717 | 2095 | 0 | 0 | 7358 |
| Cross | 2107 | 2275 | 2875 | 0 | 0 | 7258 |
| Loíza | 0 | 0 | 7123 | 0 | 0 | 7123 |
| Terrebonne | 729 | 4248 | 1991 | 0 | 0 | 6968 |
| Jenkins | 2722 | 1125 | 3069 | 0 | 0 | 6916 |
| Liberty | 1803 | 5100 | 0 | 0 | 0 | 6903 |
| Chesterfield | 637 | 3946 | 2304 | 0 | 0 | 6887 |
| Onslow | 0 | 85 | 6752 | 0 | 0 | 6837 |
| Turner | 1907 | 1396 | 3511 | 0 | 0 | 6814 |
| George | 306 | 4047 | 2457 | 0 | 0 | 6810 |
| Miller | 3434 | 3136 | 0 | 0 | 0 | 6570 |
| Blount | 0 | 1968 | 4548 | 0 | 0 | 6516 |
| Durham | 0 | 1067 | 5449 | 0 | 0 | 6516 |
| Bamberg | 616 | 2342 | 3545 | 0 | 0 | 6503 |
| Sierra | 669 | 1673 | 4156 | 0 | 0 | 6498 |
| East Carroll | 1442 | 1287 | 3736 | 0 | 0 | 6465 |
| Catoosa | 0 | 0 | 6368 | 0 | 0 | 6368 |
| Franklin | 1299 | 4989 | 14 | 0 | 0 | 6302 |
| Marengo | 2999 | 1875 | 1379 | 0 | 0 | 6254 |
| McCormick | 492 | 4844 | 903 | 0 | 0 | 6240 |
| Evans | 334 | 3204 | 2445 | 0 | 0 | 5983 |
| Klamath | 865 | 5113 | 0 | 0 | 0 | 5978 |
| Candler | 1540 | 4362 | 0 | 0 | 0 | 5902 |
| Hart | 81 | 5748 | 0 | 0 | 0 | 5829 |
| Schley | 1319 | 1638 | 2868 | 0 | 0 | 5825 |
| Treutlen | 1428 | 4383 | 0 | 0 | 0 | 5811 |
| Noxubee | 2217 | 3583 | 0 | 0 | 0 | 5800 |
| Hill | 1846 | 1662 | 2267 | 0 | 0 | 5776 |
| Boise | 1404 | 4338 | 0 | 0 | 0 | 5742 |
| Pender | 0 | 0 | 5732 | 0 | 0 | 5732 |
| Saline | 533 | 2798 | 2377 | 0 | 0 | 5708 |
| Clinch | 1424 | 4269 | 0 | 0 | 0 | 5693 |
| Conecuh | 3634 | 2053 | 0 | 0 | 0 | 5686 |
| Graham | 569 | 2961 | 2124 | 0 | 0 | 5654 |
| Winston | 496 | 3192 | 1946 | 0 | 0 | 5633 |
| Concordia | 1249 | 2344 | 2023 | 0 | 0 | 5616 |
| Comal | 0 | 2265 | 3316 | 0 | 0 | 5581 |
| Roane | 0 | 1825 | 3748 | 0 | 0 | 5573 |
| Gibson | 1332 | 3354 | 869 | 0 | 0 | 5555 |
| Flagler | 44 | 673 | 4834 | 0 | 0 | 5551 |
| San Jacinto | 0 | 4075 | 1348 | 0 | 0 | 5423 |
| Clarendon | 245 | 4877 | 293 | 0 | 0 | 5416 |
| Natchitoches | 672 | 637 | 4106 | 0 | 0 | 5415 |
| Rhea | 0 | 2811 | 2496 | 0 | 0 | 5307 |
| Johnston | 0 | 5182 | 0 | 0 | 0 | 5182 |
| Chattahoochee | 445 | 1148 | 3585 | 0 | 0 | 5178 |
| Cleburne | 0 | 2402 | 2748 | 0 | 0 | 5150 |
| Coconino | 444 | 3255 | 1413 | 0 | 0 | 5113 |
| Butts | 0 | 1303 | 3758 | 0 | 0 | 5062 |
| Woodruff | 1293 | 3755 | 0 | 0 | 0 | 5048 |
| Lafourche | 322 | 1305 | 3408 | 0 | 0 | 5034 |
| Sampson | 0 | 5009 | 0 | 0 | 0 | 5009 |
| Pierce | 1246 | 2509 | 1177 | 0 | 0 | 4932 |
| Imperial | 505 | 1531 | 2749 | 0 | 0 | 4785 |
| Taylor | 2959 | 1608 | 0 | 0 | 0 | 4567 |
| Screven | 2078 | 2474 | 0 | 0 | 0 | 4552 |
| Navarro | 760 | 1727 | 1927 | 0 | 0 | 4414 |
| Bullock | 1333 | 572 | 2475 | 0 | 0 | 4380 |
| Heard | 305 | 4055 | 0 | 0 | 0 | 4360 |
| Chickasaw | 1583 | 2736 | 0 | 0 | 0 | 4319 |
| Chelan | 610 | 1906 | 1790 | 0 | 0 | 4306 |
| Iberville | 0 | 355 | 3919 | 0 | 0 | 4274 |
| Camden | 268 | 3384 | 602 | 0 | 0 | 4254 |
| Warrick | 34 | 336 | 3714 | 0 | 0 | 4084 |
| Prairie | 1596 | 2408 | 0 | 0 | 0 | 4004 |
| Hatillo | 0 | 0 | 3992 | 0 | 0 | 3992 |
| Morehouse | 1352 | 2604 | 0 | 0 | 0 | 3956 |
| Haywood | 2458 | 1498 | 0 | 0 | 0 | 3956 |
| Smith | 33 | 1485 | 2434 | 0 | 0 | 3952 |
| Choctaw | 1644 | 2237 | 0 | 0 | 0 | 3882 |
| Pontotoc | 566 | 3294 | 0 | 0 | 0 | 3860 |
| Gordon | 0 | 774 | 3053 | 0 | 0 | 3827 |
| Benton | 611 | 3216 | 0 | 0 | 0 | 3827 |
| Plaquemines | 453 | 3327 | 0 | 0 | 0 | 3780 |
| Twiggs | 318 | 3315 | 27 | 0 | 0 | 3659 |
| Broward | 0 | 0 | 3630 | 0 | 0 | 3630 |
| Nash | 0 | 3555 | 17 | 0 | 0 | 3572 |
| Lafayette | 705 | 1584 | 1213 | 0 | 0 | 3502 |
| Gulf | 345 | 1352 | 1789 | 0 | 0 | 3486 |
| Austin | 22 | 1362 | 2079 | 0 | 0 | 3463 |
| Tate | 554 | 684 | 2176 | 0 | 0 | 3414 |
| Sharkey | 791 | 2583 | 0 | 0 | 0 | 3374 |
| Cape Girardeau | 259 | 1707 | 1391 | 0 | 0 | 3356 |
| St. James | 0 | 3118 | 202 | 0 | 0 | 3320 |
| Tensas | 828 | 2416 | 0 | 0 | 0 | 3244 |
| McDuffie | 328 | 2873 | 41 | 0 | 0 | 3242 |
| Cochise | 391 | 2787 | 0 | 0 | 0 | 3178 |
| Lyon | 0 | 1340 | 1805 | 0 | 0 | 3144 |
| Bryan | 32 | 984 | 2123 | 0 | 0 | 3139 |
| Jefferson Davis | 882 | 230 | 1983 | 0 | 0 | 3095 |
| Deschutes | 340 | 1147 | 1556 | 0 | 0 | 3043 |
| Loudon | 0 | 2744 | 294 | 0 | 0 | 3038 |
| Sevier | 240 | 1426 | 1368 | 0 | 0 | 3034 |
| Leake | 103 | 0 | 2821 | 0 | 0 | 2924 |
| Storey | 181 | 1171 | 1570 | 0 | 0 | 2922 |
| Wharton | 1220 | 1659 | 0 | 0 | 0 | 2879 |
| Abbeville | 167 | 1073 | 1633 | 0 | 0 | 2873 |
| Catawba | 0 | 2176 | 674 | 0 | 0 | 2850 |
| Alexander | 308 | 1498 | 960 | 0 | 0 | 2766 |
| Navajo | 142 | 602 | 1878 | 0 | 0 | 2622 |
| Stanly | 0 | 234 | 2367 | 0 | 0 | 2601 |
| Hancock | 343 | 1941 | 2 | 298 | 0 | 2584 |
| Daviess | 729 | 1846 | 0 | 0 | 0 | 2575 |
| Hopkins | 514 | 2009 | 0 | 0 | 0 | 2523 |
| Rapides | 484 | 1690 | 221 | 0 | 0 | 2395 |
| Echols | 472 | 1884 | 0 | 0 | 0 | 2356 |
| Adams | 777 | 1387 | 148 | 0 | 0 | 2312 |
| Wood | 0 | 2307 | 0 | 0 | 0 | 2307 |
| Red River | 829 | 1469 | 0 | 0 | 0 | 2298 |
| Kemper | 1319 | 976 | 0 | 0 | 0 | 2295 |
| Meigs | 0 | 2273 | 0 | 0 | 0 | 2273 |
| Hays | 0 | 0 | 2260 | 0 | 0 | 2260 |
| Itawamba | 240 | 2019 | 0 | 0 | 0 | 2259 |
| Evangeline | 577 | 1681 | 0 | 0 | 0 | 2259 |
| Prentiss | 219 | 2036 | 0 | 0 | 0 | 2255 |
| Elbert | 67 | 2159 | 8 | 0 | 0 | 2233 |
| McLean | 654 | 1491 | 0 | 0 | 0 | 2145 |
| Rowan | 0 | 94 | 2036 | 0 | 0 | 2130 |
| Independence | 419 | 1695 | 0 | 0 | 0 | 2114 |
| Tangipahoa | 82 | 2026 | 0 | 0 | 0 | 2108 |
| Fairfield | 259 | 1705 | 138 | 0 | 0 | 2102 |
| Horry | 0 | 51 | 2001 | 0 | 0 | 2052 |
| McNairy | 0 | 2034 | 0 | 0 | 0 | 2034 |
| Catahoula | 648 | 1359 | 0 | 0 | 0 | 2007 |
| Moore | 0 | 1986 | 0 | 0 | 0 | 1986 |
| Craven | 0 | 0 | 1984 | 0 | 0 | 1984 |
| Rains | 0 | 1952 | 7 | 0 | 0 | 1959 |
| Grenada | 475 | 1314 | 169 | 0 | 0 | 1958 |
| Pointe Coupee | 397 | 1531 | 0 | 0 | 0 | 1928 |
| McIntosh | 248 | 1668 | 0 | 0 | 0 | 1916 |
| Delta | 113 | 0 | 1736 | 0 | 0 | 1849 |
| White | 1341 | 503 | 0 | 0 | 0 | 1844 |
| Brantley | 254 | 1581 | 0 | 0 | 0 | 1835 |
| Brazoria | 4 | 1802 | 0 | 0 | 0 | 1806 |
| Newberry | 262 | 760 | 780 | 0 | 0 | 1802 |
| Falls | 453 | 1321 | 0 | 0 | 0 | 1774 |
| Ascension | 0 | 0 | 1734 | 0 | 0 | 1734 |
| West Feliciana | 110 | 1593 | 0 | 0 | 0 | 1703 |
| Cameron | 911 | 736 | 0 | 0 | 0 | 1647 |
| Gallatin | 569 | 1068 | 0 | 0 | 0 | 1637 |
| Ballard | 563 | 111 | 946 | 0 | 0 | 1620 |
| Grant | 444 | 1165 | 0 | 0 | 0 | 1608 |
| Waller | 0 | 594 | 985 | 0 | 0 | 1579 |
| Colorado | 474 | 1099 | 0 | 0 | 0 | 1573 |
| Saluda | 0 | 1555 | 6 | 0 | 0 | 1561 |
| St. Martin | 67 | 1487 | 0 | 0 | 0 | 1554 |
| Cabarrus | 0 | 1542 | 0 | 0 | 0 | 1542 |
| Neshoba | 91 | 1431 | 0 | 0 | 0 | 1522 |
| Conway | 226 | 1267 | 0 | 0 | 0 | 1493 |
| Titus | 0 | 1487 | 0 | 0 | 0 | 1487 |
| Stephens | 0 | 1475 | 0 | 0 | 0 | 1475 |
| St. Lucie | 25 | 0 | 1305 | 0 | 0 | 1330 |
| Columbus | 0 | 462 | 852 | 0 | 0 | 1314 |
| Wilkinson | 694 | 609 | 0 | 0 | 0 | 1303 |
| Angelina | 105 | 1094 | 89 | 0 | 0 | 1288 |
| Rusk | 254 | 1005 | 0 | 0 | 0 | 1259 |
| Crenshaw | 494 | 762 | 0 | 0 | 0 | 1256 |
| Vance | 0 | 1237 | 0 | 0 | 0 | 1237 |
| Gaston | 0 | 0 | 1230 | 0 | 0 | 1230 |
| Cullman | 0 | 1229 | 0 | 0 | 0 | 1229 |
| Hamblen | 0 | 1190 | 0 | 0 | 0 | 1190 |
| Stone | 620 | 562 | 0 | 0 | 0 | 1182 |
| Van Buren | 0 | 1153 | 0 | 0 | 0 | 1153 |
| St. Helena | 258 | 885 | 0 | 0 | 0 | 1144 |
| Bollinger | 292 | 811 | 0 | 0 | 0 | 1103 |
| Yakima | 487 | 577 | 0 | 0 | 0 | 1064 |
| Trigg | 146 | 914 | 0 | 0 | 0 | 1060 |
| Yalobusha | 258 | 794 | 0 | 0 | 0 | 1052 |
| Gem | 753 | 294 | 0 | 0 | 0 | 1047 |
| Long | 37 | 991 | 0 | 0 | 0 | 1028 |
| St. Mary | 32 | 987 | 0 | 0 | 0 | 1019 |
| Camp | 1 | 993 | 0 | 0 | 0 | 994 |
| Glascock | 95 | 893 | 0 | 0 | 0 | 988 |
| Apache | 0 | 962 | 0 | 0 | 0 | 962 |
| Alpine | 35 | 853 | 70 | 0 | 0 | 958 |
| Vermilion | 322 | 629 | 0 | 0 | 0 | 951 |
| Van Zandt | 0 | 627 | 313 | 0 | 0 | 940 |
| Burleson | 380 | 540 | 0 | 0 | 0 | 920 |
| Tishomingo | 280 | 637 | 0 | 0 | 0 | 917 |
| St. Landry | 337 | 579 | 0 | 0 | 0 | 916 |
| Grainger | 0 | 915 | 0 | 0 | 0 | 915 |
| Talbot | 556 | 348 | 0 | 0 | 0 | 904 |
| Chester | 0 | 821 | 0 | 0 | 0 | 821 |
| Bowie | 199 | 616 | 0 | 0 | 0 | 815 |
| Modoc | 809 | 0 | 0 | 0 | 0 | 809 |
| Bladen | 160 | 640 | 0 | 0 | 0 | 800 |
| Beauregard | 0 | 796 | 0 | 0 | 0 | 796 |
| Gilchrist | 85 | 710 | 0 | 0 | 0 | 795 |
| Carteret | 18 | 498 | 273 | 0 | 0 | 788 |
| Bienville | 345 | 439 | 0 | 0 | 0 | 784 |
| Charlton | 491 | 278 | 0 | 0 | 0 | 769 |
| Ohio | 155 | 613 | 0 | 0 | 0 | 768 |
| Towns | 0 | 761 | 0 | 0 | 0 | 761 |
| Prince George | 0 | 745 | 0 | 0 | 0 | 745 |
| Wakulla | 177 | 565 | 0 | 0 | 0 | 742 |
| Northampton | 0 | 730 | 0 | 0 | 0 | 730 |
| Le Flore | 207 | 501 | 0 | 0 | 0 | 708 |
| Dawson | 0 | 0 | 687 | 0 | 0 | 687 |
| Calloway | 153 | 523 | 0 | 0 | 0 | 676 |
| Yell | 24 | 374 | 272 | 0 | 0 | 670 |
| Isabela | 0 | 0 | 666 | 0 | 0 | 666 |
| Attala | 368 | 256 | 20 | 0 | 0 | 643 |
| Baxter | 0 | 640 | 0 | 0 | 0 | 640 |
| Meriwether | 398 | 237 | 0 | 0 | 0 | 635 |
| Mayagüez | 0 | 0 | 622 | 0 | 0 | 622 |
| Coosa | 278 | 340 | 0 | 0 | 0 | 618 |
| Graves | 0 | 554 | 0 | 0 | 0 | 554 |
| Freestone | 47 | 505 | 0 | 0 | 0 | 552 |
| Bosque | 0 | 551 | 0 | 0 | 0 | 551 |
| San Augustine | 252 | 124 | 170 | 0 | 0 | 546 |
| Spencer | 41 | 487 | 0 | 0 | 0 | 528 |
| Hampton | 72 | 430 | 0 | 0 | 0 | 503 |
| Issaquena | 337 | 151 | 0 | 0 | 0 | 489 |
| Pittsburg | 0 | 486 | 0 | 0 | 0 | 486 |
| Cataño | 0 | 0 | 471 | 0 | 0 | 471 |
| Nacogdoches | 215 | 254 | 0 | 0 | 0 | 469 |
| Skamania | 0 | 66 | 401 | 0 | 0 | 466 |
| Murray | 0 | 464 | 0 | 0 | 0 | 464 |
| Tippah | 127 | 321 | 0 | 0 | 0 | 448 |
| Caldwell | 0 | 224 | 189 | 0 | 0 | 413 |
| Dixie | 4 | 408 | 0 | 0 | 0 | 412 |
| Little River | 25 | 376 | 0 | 0 | 0 | 401 |
| Grayson | 35 | 356 | 0 | 0 | 0 | 391 |
| Sequoyah | 190 | 199 | 0 | 0 | 0 | 389 |
| Ripley | 348 | 20 | 0 | 0 | 0 | 367 |
| Cooke | 144 | 208 | 0 | 0 | 0 | 352 |
| Pickett | 18 | 326 | 0 | 0 | 0 | 344 |
| Upshur | 19 | 321 | 0 | 0 | 0 | 339 |
| Tyler | 62 | 277 | 0 | 0 | 0 | 339 |
| Barren | 161 | 175 | 0 | 0 | 0 | 336 |
| Upson | 117 | 197 | 0 | 0 | 0 | 314 |
| LaSalle | 96 | 215 | 0 | 0 | 0 | 311 |
| Logan | 219 | 24 | 61 | 0 | 0 | 304 |
| Brazos | 207 | 97 | 0 | 0 | 0 | 304 |
| James City | 0 | 293 | 0 | 0 | 0 | 293 |
| Person | 0 | 287 | 0 | 0 | 0 | 287 |
| Breckinridge | 0 | 281 | 0 | 0 | 0 | 281 |
| Muhlenberg | 36 | 231 | 0 | 0 | 0 | 267 |
| Allen | 198 | 65 | 0 | 0 | 0 | 262 |
| Hardee | 0 | 257 | 0 | 0 | 0 | 257 |
| De Soto | 255 | 1 | 0 | 0 | 0 | 257 |
| Oglethorpe | 0 | 0 | 249 | 0 | 0 | 249 |
| Milam | 247 | 0 | 0 | 0 | 0 | 247 |
| Trousdale | 0 | 236 | 0 | 0 | 0 | 236 |
| Claiborne | 52 | 178 | 0 | 0 | 0 | 231 |
| Hempstead | 39 | 189 | 0 | 0 | 0 | 228 |
| Robertson | 222 | 2 | 0 | 0 | 0 | 224 |
| McCurtain | 220 | 0 | 0 | 0 | 0 | 220 |
| Halifax | 18 | 201 | 0 | 0 | 0 | 219 |
| Whitley | 0 | 212 | 0 | 0 | 0 | 212 |
| McMinn | 101 | 110 | 0 | 0 | 0 | 211 |
| Effingham | 0 | 206 | 0 | 0 | 0 | 206 |
| St. John the Baptist | 57 | 141 | 0 | 0 | 0 | 198 |
| Weakley | 189 | 0 | 0 | 0 | 0 | 189 |
| Hardeman | 0 | 189 | 0 | 0 | 0 | 189 |
| Charles City | 0 | 177 | 0 | 0 | 0 | 177 |
| Haskell | 122 | 54 | 0 | 0 | 0 | 176 |
| Toa Baja | 0 | 0 | 175 | 0 | 0 | 175 |
| Fannin | 170 | 0 | 0 | 0 | 0 | 170 |
| Kittitas | 170 | 0 | 0 | 0 | 0 | 170 |
| Copiah | 103 | 59 | 0 | 0 | 0 | 162 |
| Morris | 160 | 0 | 0 | 0 | 0 | 160 |
| Gilmer | 0 | 157 | 0 | 0 | 0 | 157 |
| East Feliciana | 115 | 38 | 0 | 0 | 0 | 153 |
| Colleton | 3 | 141 | 0 | 0 | 0 | 144 |
| Cleveland | 141 | 0 | 0 | 0 | 0 | 141 |
| Assumption | 130 | 0 | 0 | 0 | 0 | 130 |
| Cass | 45 | 80 | 0 | 0 | 0 | 126 |
| Dillon | 124 | 0 | 0 | 0 | 0 | 124 |
| Howard | 121 | 0 | 0 | 0 | 0 | 121 |
| Anson | 110 | 0 | 0 | 0 | 0 | 110 |
| Grimes | 84 | 0 | 0 | 0 | 0 | 84 |
| Granville | 0 | 77 | 0 | 0 | 0 | 77 |
| Payette | 75 | 0 | 0 | 0 | 0 | 75 |
| Humboldt | 73 | 0 | 0 | 0 | 0 | 73 |
| Galveston | 64 | 0 | 0 | 0 | 0 | 64 |
| St. Charles | 2 | 0 | 61 | 0 | 0 | 64 |
| Hyde | 62 | 0 | 0 | 0 | 0 | 62 |
| Laurel | 0 | 60 | 0 | 0 | 0 | 60 |
| Tyrrell | 57 | 0 | 0 | 0 | 0 | 57 |
| Pamlico | 0 | 0 | 54 | 0 | 0 | 54 |
| Wilkes | 46 | 1 | 0 | 0 | 0 | 47 |
| Aguadilla | 0 | 0 | 45 | 0 | 0 | 45 |
| Clinton | 41 | 0 | 0 | 0 | 0 | 41 |
| Gloucester | 0 | 33 | 0 | 0 | 0 | 33 |
| Latimer | 32 | 0 | 0 | 0 | 0 | 32 |
| Winn | 4 | 23 | 0 | 0 | 0 | 28 |
| Sherman | 25 | 0 | 0 | 0 | 0 | 25 |
| St. Thomas | 0 | 0 | 22 | 0 | 0 | 22 |
| Muskogee | 21 | 0 | 0 | 0 | 0 | 21 |
| Chesapeake | 18 | 0 | 0 | 0 | 0 | 18 |
| Dorado | 1 | 0 | 16 | 0 | 0 | 17 |
| Iberia | 10 | 6 | 0 | 0 | 0 | 16 |
| Pushmataha | 13 | 0 | 0 | 0 | 0 | 13 |
| Christian | 10 | 0 | 0 | 0 | 0 | 10 |
| Adair | 6 | 0 | 0 | 0 | 0 | 6 |
| Coryell | 4 | 0 | 0 | 0 | 0 | 4 |
| Suffolk | 4 | 0 | 0 | 0 | 0 | 4 |
| Hopewell | 0 | 3 | 0 | 0 | 0 | 3 |
| Bedford | 0 | 1 | 0 | 0 | 0 | 1 |
| Edmonson | 0 | 0 | 0 | 0 | 0 | 0 |
